# Lactate-driven lactylation of HNRNPA1 orchestrates PKM2 splicing and glycolytic reprogramming in bladder cancer

**DOI:** 10.1186/s13046-025-03591-5

**Published:** 2025-11-22

**Authors:** Tianqi Wang, Xiaohong Ma, Yini Wang, Hongquan Liu, Guixin Ding, Yanfei Li, Hejia Yuan, Jie Gao, Fengze Sun, Yicheng Guo, Jian Ma, Jitao Wu

**Affiliations:** 1 Department of Urology, The Affiliated Yantai Yuhuangding Hospital of Qingdao University, No. 20 East Yuhuangding Road, Yantai, Shandong 264000 China; 2 Department of Urology, Shandong Provincial Hospital Affiliated to Shandong First Medical University, Jinan, Shandong China; 3 Department of Gynecology and Obstetrics, The Affiliated Yantai Yuhuangding Hospital of Qingdao University, Yantai, China; 4 The Second Clinical Medical College, Binzhou Medical University, Yantai, China

**Keywords:** HNRNPA1, Lactylation, Alternative Splicing, Glycolytic Reprogramming, Bladder Cancer

## Abstract

**Background:**

Lactylation, a recently identified post-translational modification derived from lactate, has emerged as a regulator of tumor metabolism. However, its functional relevance and molecular targets in bladder cancer (BLCA) remain unclear.

**Methods:**

We performed immunohistochemistry on patient tissues, global lactylation proteomics using LC–MS/MS, and in vitro and in vivo functional assays. Gene editing via CRISPR/Cas9, overexpression systems, and pharmacological interventions were employed to study P300-mediated HNRNPA1-K350 lactylation in driving BLCA cell aggression. Metabolomics and glycolytic flux assays were used to assess the metabolic consequences of HNRNPA1 lactylation. Molecular characterization was validated through gene expression and splicing analyses. Small-molecule drug screening was conducted via molecular docking to identify potential inhibitors targeting HNRNPA1.

**Results:**

Protein lactylation levels were significantly elevated in BLCA tissues, correlating with poor prognosis. HNRNPA1 was identified as a central lactylation target. Glycolysis-induced lactate production promoted P300-mediated lactylation of HNRNPA1 at lysine 350, which facilitated PKM pre-mRNA splicing toward the PKM2 isoform, enhancing glycolytic flux and supporting tumor growth. Inhibition of glycolysis or LDHA knockdown reduced HNRNPA1 lactylation, suppressed PKM2 expression, and impaired BLCA cell proliferation, migration, and invasion. Metabolomic profiling linked HNRNPA1-K350 lactylation with increased aerobic glycolysis in BLCA cells. A small-molecule inhibitor, identified through molecular docking, attenuated cell proliferation by binding to HNRNPA1 and suppressing PKM2 expression.

**Conclusions:**

This study reveals a lactate-driven mechanism coupling alternative splicing to metabolic reprogramming via HNRNPA1 lactylation, identifying HNRNPA1-K350 lactylation as a key driver of glycolysis-dependent tumor progression. A therapeutic approach targeting HNRNPA1 in BLCA is proposed.

**Supplementary Information:**

The online version contains supplementary material available at 10.1186/s13046-025-03591-5.

## Introduction

Bladder cancer (BLCA) stands as one of the most prevalent malignancies in the urinary system, ranking tenth in global incidence [1]. Although surgical excision remains the standard treatment modality, BLCA is often characterized by high recurrence rates and aggressive biological behavior, which contribute to unfavorable clinical outcomes [2]. Currently, there is a notable deficiency in both specific molecular diagnostic biomarkers and efficacious therapeutic approaches for BLCA. This underscores the need for deeper insights into the molecular mechanisms driving BLCA initiation and progression.

A hallmark of cancer is metabolic reprogramming, typified by the Warburg effect, the preference of cancer cells to ferment glucose into lactate even in the presence of oxygen (aerobic glycolysis) [3]. This metabolic shift allows rapidly proliferating tumor cells to meet increased bioenergetic and biosynthetic demands [4]. The accumulation of lactate, a byproduct of this enhanced glycolysis, is more than a metabolic waste. High levels of lactate in tumors are associated with invasiveness, immune evasion, and therapy resistance [5, 6]. Moreover, metabolites like lactate can serve as substrates for novel post-translational modifications (PTMs) that link metabolism to epigenetic and signaling pathways [7].

Lysine lactylation is a recently characterized PTM in which a lactate molecule is covalently attached to lysine residues. Lactate-derived histone lysine lactylation was shown to directly stimulate gene transcription as an epigenetic mark [8]. For example, histone lactylation has been shown to drive oncogenic programs in tumors. In ocular melanoma, elevated histone H3 lysine 18 lactylation upregulates the m6A reader YTHDF2, promoting degradation of tumor-suppressive mRNAs such as *PER1* and *TP53*, and thereby accelerating tumor growth [9]. While glycolysis is frequently dysregulated in BLCA, the precise role of lactylation in this context remains insufficiently characterized. Emerging evidence suggests that circular RNA *circXRN2* impedes BLCA progression by suppressing *H3K18* lactylation [10]. Beyond histones, non-histone protein lactylation is emerging as an important regulatory mechanism, although its scope and functional consequences are only beginning to be understood.

Given the highly glycolytic nature of BLCA tumors and the resulting lactate-rich microenvironment [11], we hypothesized that lactylation of non-histone proteins might play a pivotal role in BLCA progression. Here, we set out to investigate whether protein lactylation contributes to BLCA tumorigenesis and to identify key lactylated substrates and pathways involved. Through comprehensive lactyl-proteomic analysis, we discovered that heterogeneous nuclear ribonucleoprotein A1 (HNRNPA1) is a major lactylated protein in BLCA. HNRNPA1 is a ubiquitously expressed RNA-binding protein known to regulate alternative splicing and RNA metabolism [12, 13]. HNRNPA1 plays a multifaceted role in molecular pathways that drive malignant transformation [14, 15]. Importantly, the metabolic regulatory roles of HNRNPs are not fixed but are subject to dynamic modulation through PTMs. In hepatocellular carcinoma, lysine residues in HNRNPA1 were deacetylated by SIRT1 and SIRT6, resulting in significant inhibition of glycolysis [16]. Similarly, in lung adenocarcinoma, ESCO2-mediated acetylation of HNRNPA1 contributes to metabolic reprogramming and tumor progression [17]. However, the role of HNRNPA1 in BLCA metabolism remained unclear prior to our study.

In the present study, we demonstrate that lysine 350 (K350) lactylation of HNRNPA1 is significantly elevated in BLCA tissues. Functional assays revealed that either genetic knockout of HNRNPA1 or substitution of K350 with arginine (K350R) markedly suppressed both the malignant phenotypes and the aerobic glycolysis in BLCA, in vitro and in vivo. Further investigation identified P300 as the responsible lactyltransferase catalyzing this modification. Mechanistically, lactylation at HNRNPA1 K350 promotes tumor progression and metabolic reprogramming by regulating the expression of pyruvate kinase M2 (PKM2).

Collectively, these findings unveil a previously unrecognized function of non-histone protein lactylation in BLCA. Specifically, disruption of the glycolysis–HNRNPA1-K350 lactylation–PKM2 positive feedback axis represents a promising therapeutic avenue for targeting metabolic vulnerability in BLCA.

## Materials and methods

### Clinical samples

Human bladder cancer tissues and adjacent normal bladder tissues were obtained from patients undergoing radical cystectomy or transurethral resection of bladder tumor (TURBT) at our institution between September 2019 and December 2022. All samples were confirmed as urothelial carcinoma by two independent pathologists. Informed consent was obtained from each patient, and the study was approved by the Ethics Committee of the Affiliated Yantai Yuhuangding Hospital of Qingdao University. Tissue microarrays were constructed for IHC analysis. Relevant clinical data (tumor stage, grade, patient outcomes) were collected and are summarized in Supplementary Table 1.

### Cell Lines and culture

BLCA cell lines (5637, RT4, EJ-1, T24, 253 J, UM-UC-3), the immortalized normal urothelial cell line SV-HUC-1, and HEK-293 T cell were obtained from the Chinese Academy of Sciences (Shanghai, China). BLCA cell lines and HEK-293 T were cultured in RPMI-1640 medium (Gibco, USA) supplemented with 10% fetal bovine serum (FBS) (ExCell Biotech, China), and SV-HUC-1 cells were maintained in F-12 K medium (Gibco) with 10% FBS. All cell lines were incubated at 37℃ in a humidified 5% CO_2_ atmosphere. Cells were transfected once they reached approximately 30–50% confluence.

### HNRNPA1 knockout and overexpression

An HNRNPA1 knockout was created in EJ-1 and UM-UC-3 cells using CRISPR/Cas9. Single guide RNA (sgRNA) targeting HNRNPA1 was designed (sequence: 5′-GCGTTCCCCATTGCTCAAAA-3′) and cloned into lentiCRISPRv2 (Abm, Canada). Lentivirus was produced in HEK-293 T cells and used to infect BLCA cells, followed by puromycin selection. Single-cell clones were expanded and screened by western blot for loss of HNRNPA1 protein. For rescue experiments, human HNRNPA1 cDNA (NM_031157.4) was cloned into the pcSLenti-EF1-EGFP-P2A-Puro-CMV-MCS lentivector (Obio Technology, China). The K350R point mutation (AAA to AGA at the codon for lysine 350) was introduced by site-directed mutagenesis and confirmed by sequencing. Lentiviruses carrying HNRNPA1-WT or K350R were transduced into HNRNPA1-KO cells to establish stable isogenic lines. Expression was verified by western blot, and clones with comparable HNRNPA1 expression were used.

### Plasmid construction and siRNA transfection

All plasmids and siRNAs used in this study are listed in Supplementary Table 2. Overexpression plasmids for His-PKM2, His-P300, His-CBP, Flag-GCN5, and Flag-PCAF were constructed by Obio Technology and General Bio. Overexpression plasmid for Flag-HNRNPA1 was constructed by YHDURO. To introduce plasmids and siRNAs into the cell lines, X-tremeGENE HP DNA Transfection Reagent (Roche, Switzerland) was employed according to the manufacturer’s instructions.

### Drug treatment

The drugs used in this study are detailed in Supplementary Table 3. Cells were treated with NaLa (867–56-1, Sigma Aldrich) for 24 h to enhance global lactylation levels. Glycolysis was inhibited by exposing cells to oxamate (HY-W013032A, MCE) and 2-DG (HY-13966, MCE) for 24 h. P300 activation was achieved by administering CTB (HY-134964, MCE) for 24 h, while P300 inhibition was performed with C646 (HY-13823, MCE) for the same duration. Cells were treated with polydatin (27208–80-6, TargetMol, USA) for 24 h to examine cell proliferation. DMSO was used as vehicle control for drug treatments. For each treatment, cell viability and lactylation levels were monitored to ensure treatments were effective but not causing excessive cell death.

### CCK-8 and colony formation

BLCA cells were cultured in 6 cm plates and transfected with plasmids or treated with drugs for 24 h. Subsequently, cells were trypsinized and counted to determine the appropriate seeding density for subsequent assays. For the CCK-8 assay, 1,000 cells per well were seeded into 96-well plates. Cell viability was assessed using a CCK-8 assay kit (New Cell & Molecular Biotech, China) in accordance with the manufacturer’s instructions. Absorbance at 450 nm was measured using a microplate reader at 0, 24, 48, 72, 96, and 120 h.

To determine drug IC₅₀ values, 8,000 cells were seeded into 96-well plates and treated with increasing concentrations of the drug for 24 h. Cell viability was then measured using the same CCK-8 protocol.

Colony formation assays were conducted by seeding cells in 6-well plates (1000 cells per well) and allowing 7–10 days for colony growth. Colonies were fixed with methanol and stained with crystal violet (Beyotime, China) and stained colonies were imaged and quantified for statistical analysis.

### EdU assay

Cell proliferation was assessed using an EdU Cell Proliferation Kit (Ribobio, China). SV-HUC-1 and BLCA cells (3,000 cells per well) were seeded in 96-well plates and incubated overnight at 37 ℃. SV-HUC-1 cells were treated with 0.1% DMSO (control), 10 mM NaLa, or 20 mM NaLa for 24 h. Following treatment, cells were incubated with EdU reagent for 2 h, then fixed with methanol for 15 min. After permeabilization with 0.4% Triton X-100 (Servicebio, China) for 10 min, cells were incubated with 100 μl Apollo® fluorescent dye for 30 min. Nuclei were stained with Hoechst for 30 min, and images were acquired using an Olympus inverted fluorescence microscope. Proliferation was quantified by calculating the ratio of EdU-positive (red) to Hoechst-positive (blue) cells.

### In vivo tumorigenesis assay

Male BALB/c nude mice aged four weeks were sourced from SLAC Laboratory Animal Co. (Shanghai, China) and randomly allocated to different experimental groups (n = 5 mice per group). A total of 1 × 10⁷ cells suspended in 100 μl serum-free RPMI 1640 medium were subcutaneously injected into the right flank of each mouse. For the lactate supplementation experiment, EJ-1 (parental) cells were injected (1 × 10⁷ per mouse) and, starting on day 4 post-injection, mice were given daily intraperitoneal injections of NaLa (150 mg/kg) or vehicle (saline) for 14 consecutive days. For polydatin therapy, mice were treated with 25 mg/kg polydatin or DMSO (intratumoral injection, every other day for 2 weeks). Tumor dimensions were measured every 3–4 days with calipers, and volumes were calculated as (length × width^2^)/2. Two weeks after cell injection, mice were euthanized, and xenograft tumors were collected for subsequent analyses.

### Wound healing assay

A total of 8 × 10^5^ cells were seeded into 6-well plates and cultured until reaching full confluence. Linear wounds were created by scratching the cell monolayer with a sterile 1,000 μl pipette tip. Detached cells were removed by washing twice with PBS, and fresh basal medium was added. Images of the wound area were captured at 0 and 18 h using an inverted microscope (Olympus, Japan). The extent of cell migration was quantified by measuring the wound closure area using Image J software.

### Transwell invasion assay

For the invasion assay, 5 × 10^4^ cells were seeded in the upper chamber of a Transwell insert (Corning, USA), pre-coated with Matrigel if required, in serum-free medium. The lower chamber was filled with medium containing 10% FBS to serve as a chemoattractant. After 24 h of incubation at 37 ℃ in a 5% CO₂ atmosphere, the non-invading cells on the upper surface were removed with a cotton swab. Invaded cells on the lower surface were fixed, stained with crystal violet for 20 min, and visualized under a microscope.

### Cellular apoptosis evaluation via flow cytometry

To assess apoptosis, cells were treated with 0.1% DMSO (control), 20 mM oxamate, or 2-DG for 48 h. After treatment, 5 × 10^5^ cells were collected, washed, stained with FITC Annexin V and propidium iodide (PI)(40302ES, Yeason, China), and gently vortexed. Samples were incubated at room temperature for 20 min in the dark. Apoptotic cells were analyzed using a FACS flow cytometer (Beckman Coulter, USA) in accordance with the manufacturer’s instructions. Data were processed and quantified using FlowJo software.

### Western blot analysis

Protein lysates were separated via SDS-PAGE and transferred onto polyvinylidene difluoride (PVDF) membranes. Following a blocking step with an appropriate blocking solution, membranes were incubated with specific primary antibodies overnight at 4 ℃. Afterward, membranes were treated with corresponding horseradish peroxidase-conjugated secondary antibodies for 1 h at room temperature. Membranes were washed three times for 10 min each in TBST. Protein bands were visualized using an enhanced chemiluminescence (ECL) detection system. A comprehensive list of primary and secondary antibodies used is provided in Supplementary Table 4.

### Co-IP assay

HEK-293 T, EJ-1, and UM-UC-3 cells, along with BLCA tissue samples, were lysed in 500 μl of IP lysis buffer (New Cell & Molecular Biotech, China) supplemented with phenylmethylsulfonyl fluoride (PMSF). Lysates were centrifuged at 12,000 × g for 10 min at 4 ℃. Supernatants were incubated with the appropriate antibody and Protein A/G agarose beads (Santa Cruz, USA) overnight at 4 ℃ with gentle rotation. Beads were subsequently washed six times with wash buffer. The bound proteins were then eluted and analyzed by western blotting as described above.

### Site-specific antibody production

A rabbit polyclonal antibody against HNRNPA1 lactylated at K350 was custom-generated (PTM Bio, China). Briefly, a synthetic peptide encompassing the HNRNPA1 sequence around K350 with a lactylated lysine modification was used to immunize rabbits. The antibody was affinity-purified using the lactylated peptide, with a parallel purification against the non-modified peptide to ensure specificity. Specificity was validated by dot blot and western blot: the antibody recognized lactylated but not non-lactylated HNRNPA1 peptide, and it detected a band at the expected size of HNRNPA1 in WT but not K350R mutant cell lysates.

#### IHC

According to previous study [18], IHC was carried out. Nuclei in immunohistochemistry were counterstained with Hematoxylin (Solarbio, China). For tissue microarray analysis, immunohistochemical scoring of Pan-lactylation and P300 was calculated by multiplying the staining intensity score by the percentage of positively stained cells. A full list of antibodies used is available in Supplementary Table 4.

### qPCR

Total RNA was extracted from cells using Trizol reagent (Thermo Fisher Scientific, USA) in accordance with the manufacturer’s protocol. The isolated RNA was reverse-transcribed into single-stranded complementary DNA (cDNA) using a cDNA Synthesis Kit (ACCURATE BIOTECHNOLOGY (HUNAN) CO.,LTD, China). qPCR was carried out using SYBR Green Master Mix (ACCURATE BIOTECHNOLOGY (HUNAN) CO.,LTD, China) on a real-time PCR detection system. Primer sequences used in this study are listed in Supplementary Table 5.

### Trypsin digestion, enrichment of PTM Peptides and LC–MS/MS analysis

Detailed procedure is provided in the Supplementary Materials.

### Metabolite measurements

For metabolomic profiling, the detail is provided in the Supplementary Materials. Targeted quantification of glycolytic intermediates, lactate, ATP, ADP, NADPH, NADP^+^ was performed using multiple reaction monitoring (MRM) on a triple quadrupole mass spectrometer. Standard curves of authentic metabolites were used for quantification. Glucose uptake was measured using a glucose uptake colorimetric kit (Abcam, UK) in accordance with the manufacturer’s instructions. Intracellular lactate concentration was measured with a Lactate Assay Kit (Abcam, UK). Glucose content was determined according to the manufacturers instruction (Boxbio Science&Technology, China). ATP levels in cells were determined using an ATP Determination Kit (Beyotime Biotechnology, China), and NADP^+^/NADPH and GSH/GSSG ratios were measured with assay kits from Beyotime Biotechnology, following the manufacturers’ protocols.

### Extracellular Acidification Rate (ECAR)

ECAR was measured using the Seahorse XF Glycolysis Stress Test (Agilent Technologies). Cells (4 × 10^4^ per well) were seeded in XF24 microplates and incubated overnight. On the day of assay, cells were equilibrated in Seahorse assay medium, and ECAR was recorded at baseline and after sequential injections of glucose (10 mM), oligomycin (1 μM), and 2-DG (50 mM). Basal glycolysis and glycolytic capacity were calculated according to manufacturer’s instructions.

### Single-cell sequencing and Gene Set Enrichment Analysis (GSEA)

Single-cell RNA sequencing datasets derived from eight BLCA tissues and three normal bladder tissues were obtained from the Genome Sequence Archive (GSA, https://ngdc.cncb.ac.cn/gsa/) under accession number HRA000212 [19]. Integration, normalization, and clustering of the datasets were performed using the Seurat (v4.0) package. Cell populations corresponding to BLCA and normal urothelial subtypes were identified through unsupervised clustering and marker gene expression profiling. The lactylation-related genes (LRGs) were defined as direct enzymatic regulators (writers/erasers), lactate producers and transporters [20]. A total of 26 LRGs were included and are presented in Supplementary Table 6. GSEA was conducted to evaluate differences in glycolysis, pyruvate metabolism, and lactic acid metabolism pathways across subpopulations. Pathway annotations were retrieved from Gene Ontology (GO), Kyoto Encyclopedia of Genes and Genomes (KEGG), and WikiPathways databases.

### Receptor-based virtual screening for HNRNPA1 inhibitors

We employed a receptor-based virtual screening approach to identify potential drug molecules that bind to the HNRNPA1 protein and inhibit its interaction with RNA. Approximately 2,300 FDA-approved drug molecules were downloaded from the DrugBank database to serve as the compound library for virtual screening. All compounds were desalted to prepare for subsequent docking simulations. The crystal structure of HNRNPA1 protein (PDB ID: 4YOE) was obtained from the RCSB PDB database (https://www.rcsb.org/structure/4YOE), and after removing water molecules and adding hydrogens, it was used as the receptor for docking simulations. All docking simulations were performed using the GPU-accelerated version of AutoDock4 [21]. The Lamarckian genetic algorithm and empirical free energy scoring function were applied for the docking process. The RNA binding pocket of HNRNPA1 was selected as the target site for the virtual screening docking simulations [22]. Flexible docking was employed, where residues within a 5 Å radius of the receptor pocket center, as well as the ligand, were set to be flexible. Each compound underwent 50 runs of genetic algorithms, and the docking conformation with the highest score for each compound was selected as the output. The compounds were then ranked according to their docking scores.

### Statistical analysis

All data represented in the figures with error of mean (mean ± SD). Statistical analyses were performed using GraphPad Prism 8. Comparisons between two groups were made using two-tailed Student’s *t*-tests, while one-way analysis of variance (ANOVA) was used for comparisons involving more than two groups. Associations between Pan-Kla levels and clinicopathological parameters were evaluated using the Chi-square test or Fisher’s exact test, as appropriate. Survival outcomes were analyzed using the Kaplan–Meier method, and statistical differences between survival curves were assessed using the log-rank test. A *p*-value less than 0.05 was considered statistically significant.

## Results

### Global lactylation is elevated in bladder cancer and correlates with poor prognosis

**Fig. 1:**
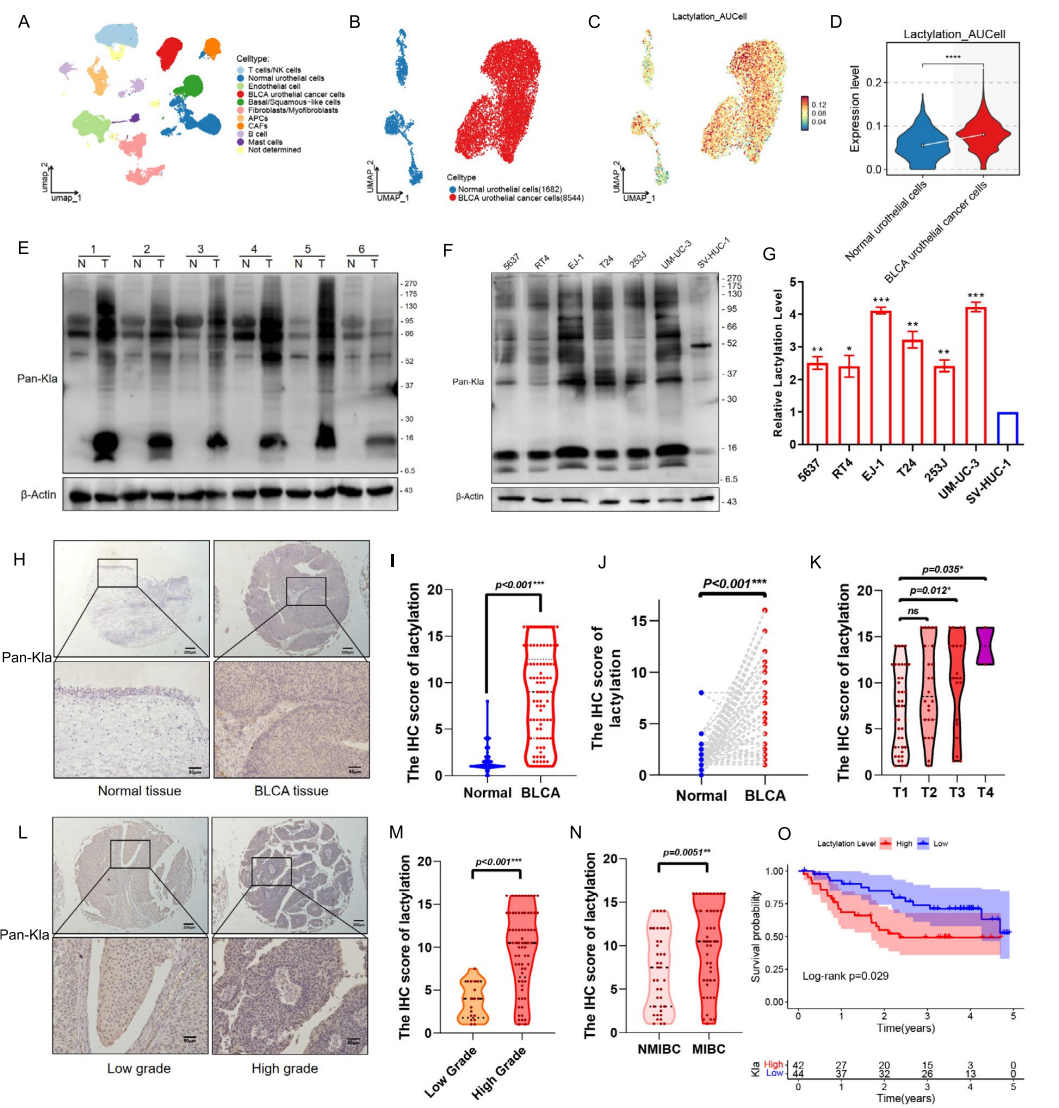
Lactylation level was increased in bladder cancer and associated with poor survival in patients with bladder cancer. **A** UMAP visualization of single-cell RNA-seq data identified by cell type. **B-C** UMAP plots, color-coded for the expression of lactylation-related genes in normal cells and bladder urothelial cells. **D** Gene-expression levels of lactylation-related genes. **E** Western blot analysis of global lactylation level in six pairs of normal tissue (N) and bladder cancer tissue (T). **F** Western blot analysis of global lactylation level in normal bladder urothelial cell SV-HUC-1 and bladder cancer cells. **G** Densitometric analysis was performed to quantify and statistically compare global lactylation levels in SV-HUC-1 and bladder cancer cell lines that normalized to β-Actin. **H** Global lactylation levels in normal and tumor tissues were visualized by immunohistochemical staining. Scale bar: 200 μm (panel above), 50 μm (lower panel). **I-J** Statistical results of lactylation levels in normal and tumor tissues. Unpaired t -test in I, paired t-test in J. (K) Lactylation levels in patients at AJCC stages T1 to T4. **L** Global lactylation levels in low-grade and high-grade tumor tissues were visualized by immunohistochemical staining. Scale bar: 200 μm (panel above), 50 μm (lower panel). (M) Statistical results of lactylation levels in low-grade and high-grade tumor tissues. Unpaired t-test. **N** Statistical results of lactylation levels in non-muscle-invasive bladder cancer and muscle-invasive bladder cancer tissues. Unpaired t-test. **O** Kaplan–Meier curves for overall survival between bladder cancer patients with low and high lactylation levels. *p < 0.05, **p < 0.01, ***p < 0.001

To reveal the intrinsic structure and metabolic level of the overall BLCA cells, we performed unsupervised clustering of all BLCA cells using spectral clustering (Fig. 1A). Lactylation-related genes were significantly higher in BLCA urothelial cancer cells, relative to normal urothelial cells (Fig. 1B-D). Meanwhile, GSEA revealed that BLCA cell clusters exhibited significant enrichment of metabolic pathways, including glycolysis, glycolytic process, pyruvate metabolic process, and monocarboxylic acid metabolic process (Supplementary Fig. 1A–E), indicating that BLCA cells undergo a metabolic conversion toward glycolytic process and lactate metabolism. Western blot analysis revealed significantly increased lactylation levels in BLCA tissues compared to adjacent non-tumorous tissues (Fig. 1E). Similarly, BLCA cell lines showed higher basal lactylation levels compared to the normal urothelial cell line SV-HUC-1 (Fig. 1F), consistent with the heightened glycolytic metabolism of cancer cells. Densitometric quantification confirmed a significant increase in lactylation in all tested BLCA cell lines relative to SV-HUC-1 (Fig. 1G).

To localize lactylation within tissues, we performed immunohistochemical (IHC) staining for lactylated lysine on BLCA clinical specimens. Tumor tissues showed strong nuclear and cytoplasmic lactylation signals, whereas normal urothelial tissues had minimal staining (Fig. 1H, Supplementary Table 1). Both unpaired and paired comparisons demonstrated significantly increased IHC scores for lactylation in BLCA tissues versus matched adjacent tissues (Fig. 1I-J; *p* < 0.001). We further stratified lactylation levels by tumor stage and grade. Notably, advanced stage tumors had higher lactylation than early stage tumors (Fig. 1K), and high-grade tumors showed greater lactylation than low-grade tumors (Fig. 1L-M). Similarly, muscle-invasive bladder cancers displayed higher lactylation levels than non-muscle-invasive tumors (Fig. 1N). These data indicate that lactylation elevation is associated with more aggressive disease features. Kaplan–Meier analysis showed that patients with high lactylation had significantly worse overall survival than those with low lactylation (Fig. 1O). BLCA is characterized by increased protein lactylation, and this increase correlates with higher histological grade, advanced stage, and poorer patient outcomes.

### Lactate-driven protein lactylation promotes bladder cancer cell proliferation and tumor growth

**Fig. 2:**
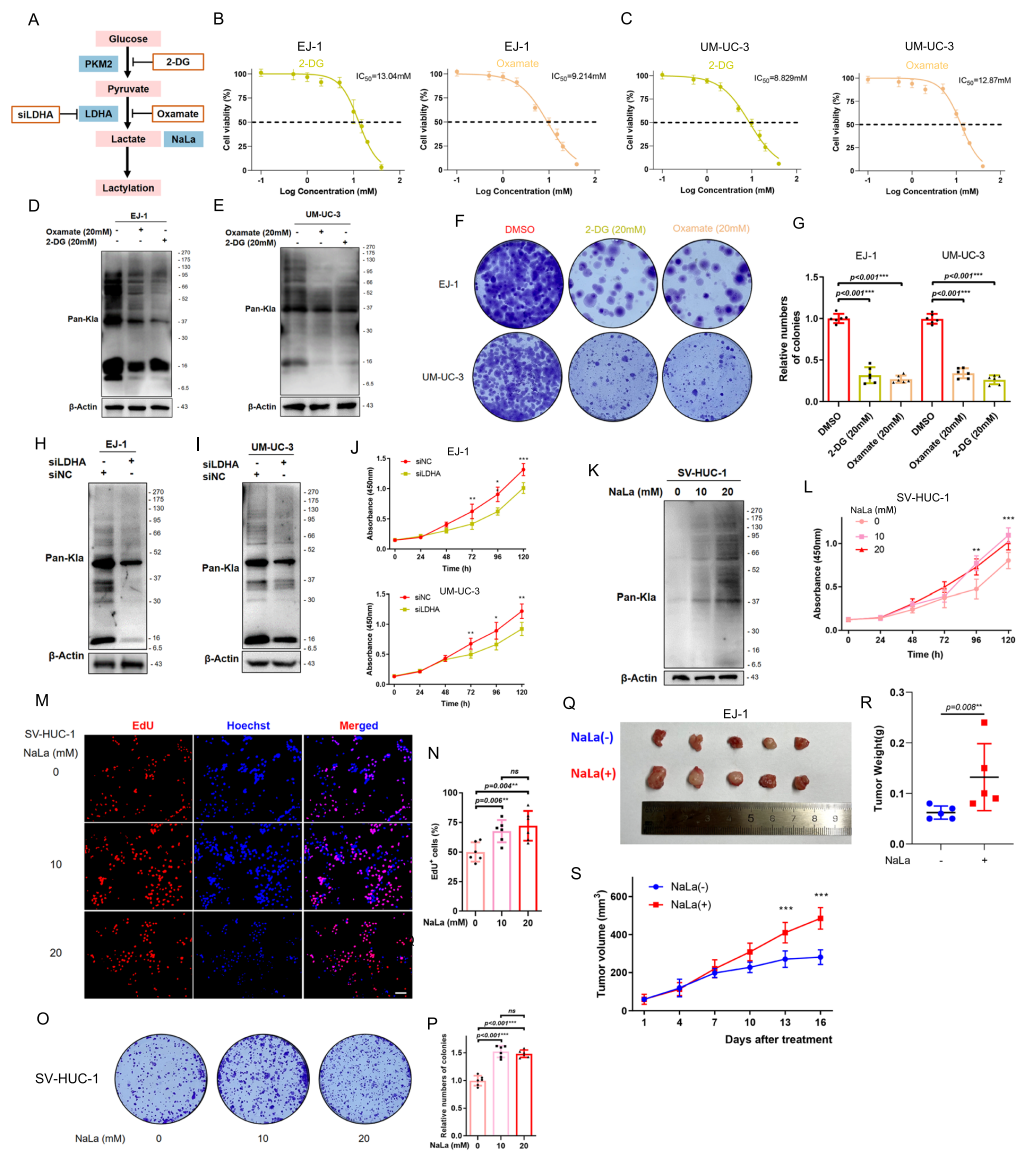
Protein lactylation facilitates tumorigenesis in bladder cancer. **A** Schematic diagram of protein lactylation inhibition methods target. **B-C** The IC_50_ values to 2-DG or oxamate in the cell lines was determined by CCK8. (D-E) Lactylation level was detected in EJ-1 and UM-UC-3 cells cultured in 20 mM 2-DG or oxamate for 24 h by western blot. **F-G** Colonial formation ability of EJ-1 and UM-UC-3 cells after adding 2-DG or oxamate was examined by the clone formation assay. (H-I) Global lactylation levels were detected in EJ-1 and UM-UC-3 cells after LDHA silencing by western blot. **J** Proliferation of EJ-1 and UM-UC-3 cells after LDHA silencing was analyzed using CCK-8 assay. **K** Lactylation level was detected in SV-HUC-1 cell cultured with or without sodium lactate (NaLa) for 24 h by western blot. **L** Proliferation of SV-HUC-1 cell treated with NaLa was analyzed using CCK-8 assay. **M-P** Cell proliferation abilities were evaluated by EdU and clone formation assays in SV-HUC-1 cell treated with NaLa. **Q-R** Nude mice were injected with EJ-1 cells subcutaneously and were intraperitoneally administered NaLa (150 mg/kg, daily) or vehicle for 14 days. Tumors were harvested on Day 16 after subcutaneous implantation. Tumor gross image and tumor weight after sacrifice. Statistical analyses were performed with unpaired two-tailed Student’s t-test (*n* = 5). (S) Tumor volume assessment during the experiment. All data are presented as mean ± SD. *p < 0.05, **p < 0.01, ***p < 0.001

We next explored whether elevated lactylation functionally contributes to BLCA tumorigenicity. To test this, we employed both pharmacological and genetic strategies to modulate lactate production and thus protein lactylation (Fig. 2A). First, we treated BLCA cells with inhibitors of glycolysis to reduce lactate levels. 2-Deoxyglucose (2-DG), a glucose analog that inhibits glycolysis, and oxamate, an inhibitor of lactate dehydrogenase, were used to treat EJ-1 and UM-UC-3 cells. Cell viability and IC_50_ values were determined using CCK8 assay (Fig. 2B-C). Western blots confirmed that both 2-DG and oxamate (20 mM, 24 h) markedly reduced global lactylation in these cells (Fig. 2D-E). Consistently, colony formation assays showed that prolonged treatment with 2-DG or oxamate dramatically reduced the clonogenic growth of BLCA cells (Fig. 2F-G). Moreover, apoptotic activity increased in response to lactylation inhibition (Supplementary Fig. 2A–B). Next, we used a genetic approach by silencing lactate dehydrogenase A (LDHA), a key enzyme that generates lactate from pyruvate. siRNA-mediated LDHA knockdown led to decreased lactylation levels in EJ-1 and UM-UC-3 cells (Supplementary Fig. 2C-D, Fig. 2H-I). This was accompanied by a significant reduction in cell proliferation, as evidenced by CCK-8 assays (Fig. 2J). Silencing LDHA further confirmed that reduced lactylation levels were associated with diminished migration and invasion capacities in BLCA cells (Supplementary Fig. 2E–G).

We then asked if increasing lactylation in non-malignant cells could enhance their proliferation. Normal SV-HUC-1 urothelial cells were treated with exogenous sodium lactate (NaLa, 10 and 20 mM). NaLa elevated global lactylation in SV-HUC-1 cells (Fig. 2K) and notably increased their proliferation rate (Fig. 2L). Further, NaLa-treated SV-HUC-1 cells formed more colonies and incorporated more EdU, indicating an increase in DNA synthesis, compared to untreated controls (Fig. 2M–P). These results imply that forcing lactylation up in normal cells can push them towards a more proliferative, tumor-like phenotype. Finally, we evaluated the effect of lactate supplementation in an in vivo setting. The result found that lactate-treated mice developed larger tumors than controls. Tumor weights at endpoint were higher in the NaLa group (Fig. 2Q-R), and tumor growth curves showed an accelerated increase in volume with lactate supplementation (Fig. 2S). Collectively, these findings support the result that protein lactylation plays a functional role in promoting tumorigenesis in BLCA, both in vitro and in vivo.

### HNRNPA1 K350 lactylation is elevated in bladder cancer tissues

**Fig. 3:**
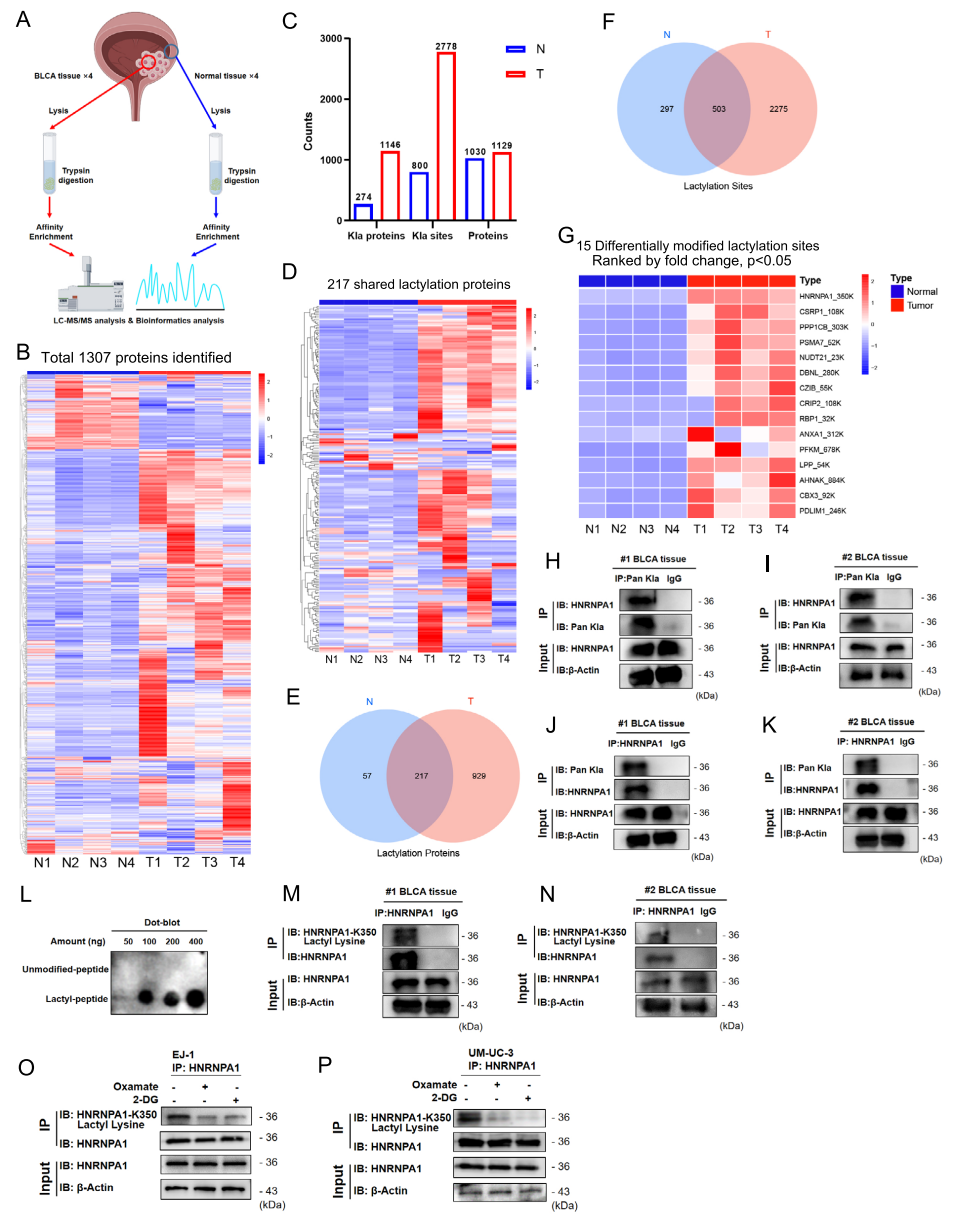
HNRNPA1 K350 lactylation is elevated in bladder cancer. **A** Flow chart showing LC–MS/MS analysis and bioinformatic analysis of bladder cancer tissues (T) and adjacent normal tissues (N) (n = 4). **B** Heatmap of total 1307 expressed proteins of N and T group samples, displayed individually for each biological replicate. Rows represented individual proteins detected by quantitative MS/MS. **C** Column graph of omics data showing the number of proteins, lactylation-modified proteins and lactylation sites identified by MS/MS. **D** Heatmap of 217 shared lactylation proteins in the N and T samples, displayed individually for each biological replicate. Rows represented individual proteins detected by quantitative MS/MS. **E** Venn diagram showing the shared and specific lactylation-modified proteins in N and T groups. **F** Venn diagram showing the shared and specific lactylation sites. **G** 15 differentially modified lactylation sites of the shared 503 lactylated modified sites, using cut offs of fold change > 1.5 and P value < 0.05. The selected 15 lactylated modified sites were ranked by fold change. **H-I** Two independent bladder cancer samples were lysed and immunoprecipitated using anti-Pan Kla antibody or control IgG followed by detection of HNRNPA1. **J-K** Two independent bladder cancer samples were lysed and immunoprecipitated using anti-HNRNPA1 antibody or control IgG followed by detection of Pan Kla. **L** Specificity of anti-HNRNPA1-K350 lactylation antibody revealed by dot-blot. (M–N) Two independent bladder cancer samples were lysed and immunoprecipitated using anti-HNRNPA1 antibody or control IgG followed by detection of HNRNPA1-K350 lactyl lysine. **O-P** EJ-1 and UM-UC-3 cells were lysed and immunoprecipitated using anti-HNRNPA1 antibody followed by detection of HNRNPA1-K350 lactyl lysine

Having established a role for lactylation in BLCA, we turned to identify the key lactylated proteins and pathways involved. We performed quantitative proteomics to profile lactylated proteins in BLCA tissues versus normal bladder tissues. Four BLCA tumors and matched adjacent normal tissues were analyzed by LC–MS/MS after immunoenrichment of lactyl-lysine containing peptides (Fig. 3A and Supplementary Fig. 3). This unbiased proteomic screen identified 1307 total proteins (Fig. 3B). In the normal tissue group, we identified 274 lactylated proteins encompassing 800 distinct lactylation sites. In contrast, the BLCA tissue group exhibited a marked increase, with 1,146 lactylated proteins and 2,778 modification sites detected (Fig. 3C). Among these, 217 lactylated proteins (Fig. 3D–E) and 503 lactylation sites (Fig. 3F) were common to both groups. Functional enrichment analysis of differentially lactylated proteins indicated significant associations with glucose metabolism and monosaccharide catabolic processes (Supplementary Fig. 4).

We next sought lactylation sites that were significantly upregulated in cancer. Using a cutoff of > 1.5-fold change in lactylation level (tumor vs normal) and *p* < 0.05, we identified 15 lactylation sites that were significantly enriched in BLCA (Fig. 3G). Strikingly, one of the top-ranked sites was lysine 350 on HNRNPA1. This site showed a high fold increase in lactylation in tumors compared to normal tissues, suggesting HNRNPA1-K350 as a candidate functionally relevant lactylation site in BLCA (Fig. 3G and Supplementary Fig. 5A–B).

To validate HNRNPA1 lactylation in vivo, we confirmed its modification in BLCA tissues, consistent with proteomic findings (Fig. 3H–K). We further developed a site-specific antibody targeting lactylated HNRNPA1 at K350 (Fig. 3L), which successfully detected K350 lactylation in BLCA samples (Fig. 3M–N). Using CRISPR/Cas9 gene editing, we knocked out HNRNPA1 in EJ-1 and UM-UC-3 cells to serve as a null background. Into these HNRNPA1-null cells, we reintroduced either wild-type HNRNPA1 (WT) or a lactylation-deficient mutant where lysine 350 was substituted with arginine (K350R). As expected, NaLa treatment failed to enhance lactylation of the K350R mutant (Supplementary Fig. 5C-D), whereas inhibition of glycolysis via oxamate and 2-DG reduced HNRNPA1-K350 lactylation (Fig. 3O–P). Additionally, the K350 residue and its surrounding sequence are evolutionarily conserved across multiple species, including *Homo sapiens*, *Mus musculus*, *Bos taurus*, and *Rattus norvegicus* (Supplementary Fig. 5E), supporting its functional relevance and selection as the focus of this study.

HNRNPA1 is a multifunctional RNA-binding protein involved in splicing regulation, RNA stability, and translation. Its identification as a prominent lactylation target raises the question of what functional role this modification might have. We hypothesized that HNRNPA1-K350 lactylation could influence its activity in regulating gene expression programs relevant to cancer progression. We therefore focused our subsequent experiments on uncovering the effects of HNRNPA1 lactylation on BLCA cell phenotype and metabolism.

### HNRNPA1-K350 lactylation promotes BLCA progression

To elucidate the functional role of HNRNPA1 K350 lactylation in BLCA progression, we employed CRISPR-Cas9 gene editing to generate HNRNPA1-knockout BLCA cell lines (Supplementary Fig. 6 A). Knockout of HNRNPA1 resulted in a marked reduction in cell viability and colony-forming capacity (Supplementary Fig. 6B–D). Additionally, HNRNPA1 depletion significantly impaired the migratory and invasive potential of BLCA cells compared to control cells (Supplementary Fig. 6E–F).

**Fig. 4:**
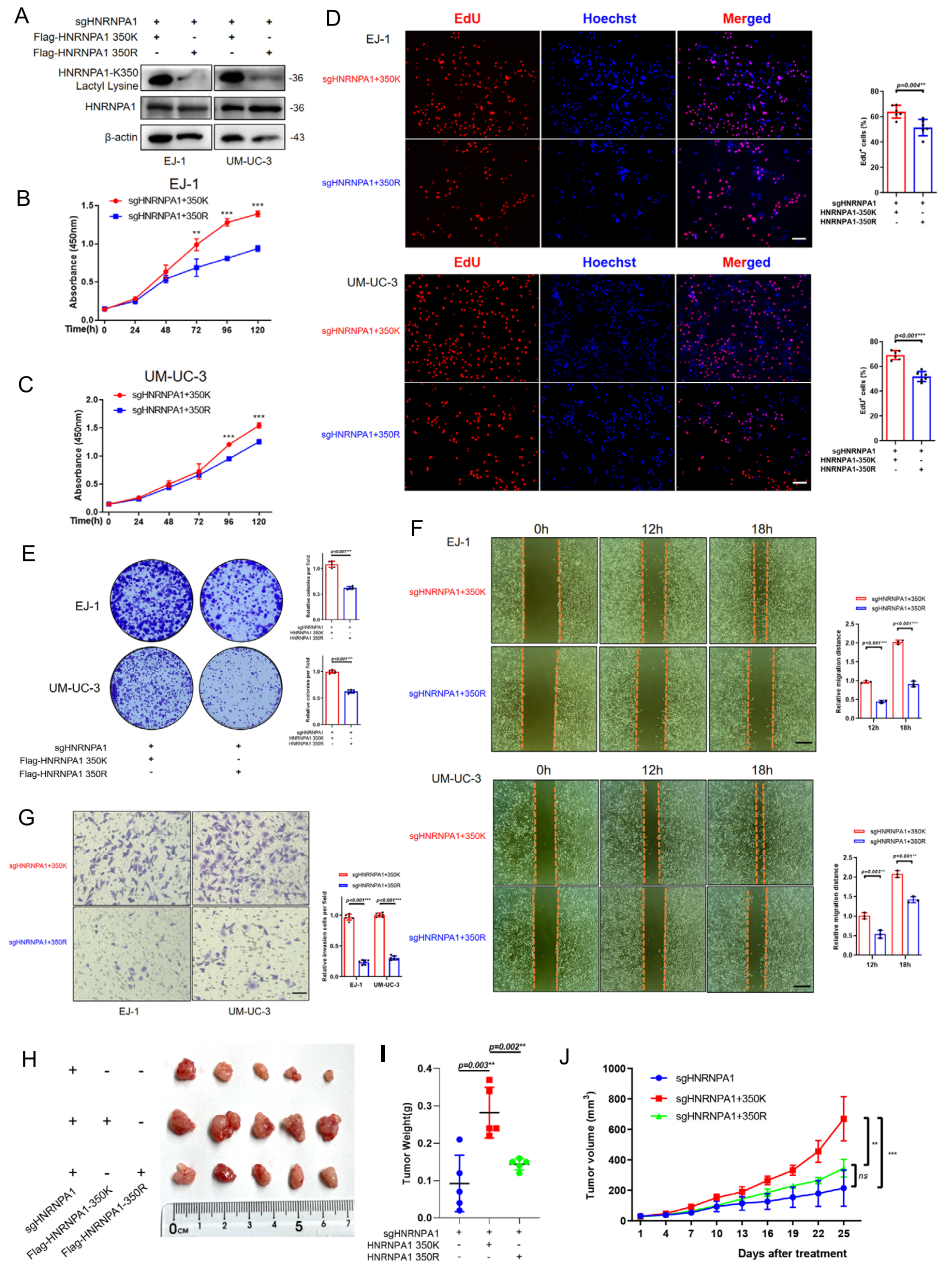
HNRNPA1-K350 lactylation promotes bladder cancer progression. **A** HNRNPA1-K350 lactyl lysine was detected by western blot in EJ-1 and UM-UC-3 cells. (B-E) Proliferation and growth of EJ-1 and UM-UC-3 cells after HNRNPA1-K350 lactylation inhibition were analyzed using CCK8 (**B-C**), EdU (**D**) and clone formation assays (**E**). **F** Migration of EJ-1 and UM-UC-3 cells after HNRNPA1-K350 lactylation inhibition was assessed using wound healing, scale bar: 200 μm. **G** Invasion of EJ-1 and UM-UC-3 cells after HNRNPA1-K350 lactylation inhibition was assessed using Transwell invasion assay, scale bar: 20 μm. (H-I) Nude mice were injected with EJ-1 cells subcutaneously. Tumors were harvested on Day 25 after subcutaneous implantation. Tumor gross image and tumor weight after sacrifice. Statistical analyses were performed with one-way ANOVA (*n* = 5). **J** Tumor volume assessment during the experiment. All data are presented as mean ± SD. *p < 0.05, **p < 0.01, ***p < 0.001

Western blotting with the anti–K350-lactylated HNRNPA1 antibody confirmed that cells expressing HNRNPA1-WT showed a robust band corresponding to K350-lactylation, whereas cells expressing HNRNPA1-K350R had no detectable signal (Fig. 4A). CCK-8 viability assays and EdU incorporation assays demonstrated that the K350R mutant had a markedly reduced proliferative capacity relative to WT (Fig. 4B–D). Colony formation assays further illustrated that HNRNPA1-WT cells formed numerous large colonies, whereas HNRNPA1-K350R cells formed notably fewer and smaller colonies (Fig. 4E). We observed similar trends in cell motility assays. In wound-healing scratch assays, BLCA cells expressing HNRNPA1-WT closed the wound area faster than K350R-expressing cells, implying greater migratory capacity (Fig. 4F). Likewise, in Transwell invasion assays, HNRNPA1-WT cells invaded through Matrigel more effectively than K350R mutant cells (Fig. 4G). Thus, HNRNPA1 promotes not only proliferation but also migration and invasion in BLCA, and these pro-metastatic functions are attenuated when lactylation at K350 is blocked. In vivo, xenograft tumor models, both HNRNPA1 knockout and selective inhibition of K350 lactylation led to a substantial decrease in tumor growth, as evidenced by reduced tumor weight and volume (Fig. 4H–J).

Together, these data underscore that HNRNPA1’s protumor functions in BLCA critically depend on its lactylation at K350. Loss of this single PTM greatly diminishes HNRNPA1’s ability to support cancer cell proliferation, migration, invasion, and tumorigenicity. This suggests that HNRNPA1-K350 lactylation is a pivotal driver of the aggressive cancer cell phenotype.

### HNRNPA1-K350 lactylation enhances aerobic glycolysis in BLCA cells

Our results so far indicated that HNRNPA1 lactylation fuels tumor growth, prompting us to investigate the underlying mechanisms. Given the known role of HNRNPA1 in regulating gene expression and splicing, and the link between lactate metabolism and lactylation, we hypothesized that HNRNPA1-K350 lactylation might alter cellular metabolism, specifically, the balance between glycolysis and oxidative phosphorylation. We therefore examined the metabolic profiles of BLCA cells with or without HNRNPA1 lactylation.

**Fig. 5:**
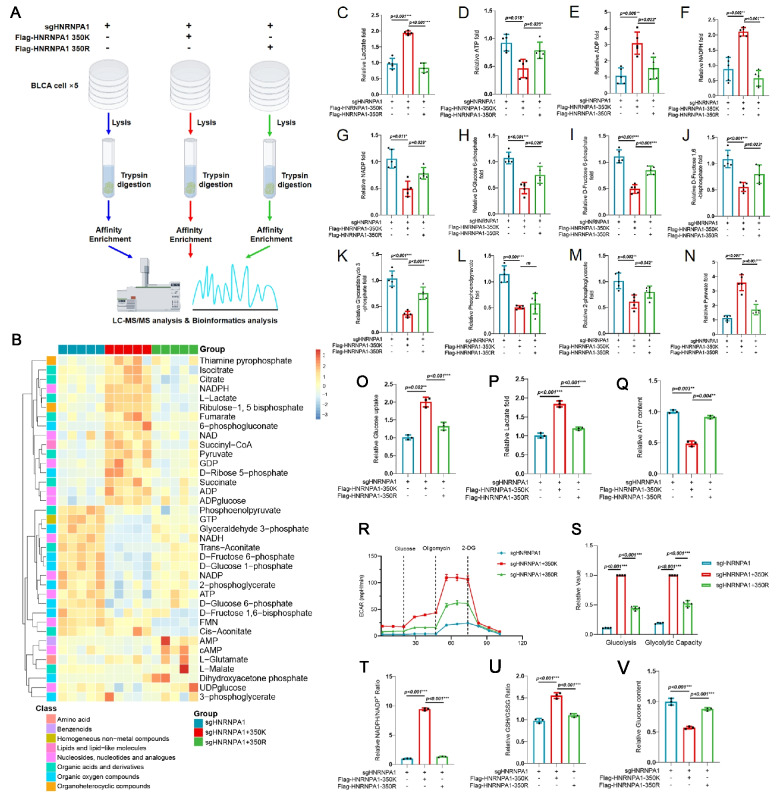
HNRNPA1-K350 lactylation enhances aerobic glycolysis in bladder cancer cells. **A** Flow chart showing LC–MS/MS analysis and bioinformatic analysis of three groups of bladder cancer cells (5 plates per group). **B** Heatmap illustrates the heterogeneity in metabolite levels across different groups. **C-N** The relative levels of metabolite in different groups were quantified by LC–MS/MS. **O-P** Glucose uptake and lactate production were detected. **Q** ATP content was determined by ATP assay kit in EJ-1 cells. **R-S** Extracellular acidification rate assay to detect basal glycolysis and glycolytic capacity. **T** NADPH/NADP^+^ assay to detect alterations of NADPH/NADP.^+^ in three types of cells (**U**) GSH/GSSG assay to detect GSH/GSSG ratio. **V** Glucose content assay to determine the relative intracellular glucose content. All data are presented as mean ± SD. *p < 0.05, **p < 0.01, ***p < 0.001

Using targeted metabolomics, we compared three groups of EJ-1 cells: HNRNPA1-knockout (HNRNPA1-KO), HNRNPA1-WT rescue, and HNRNPA1-K350R rescue. Cells were cultured under identical conditions, and polar metabolites were extracted and quantified by liquid chromatography–tandem mass spectrometry (LC–MS/MS) (Fig. 5A). Unsupervised clustering of metabolite levels revealed distinct profiles for each group, with the HNRNPA1-WT cells showing a metabolic signature indicative of heightened glycolysis (Fig. 5B).

Quantitative analysis using liquid chromatography–tandem mass spectrometry (LC–MS/MS) revealed that, relative to the knockout group, HNRNPA1-WT cells exhibited increased lactate levels (Fig. 5C), reduced ATP levels, and elevated ADP levels (Fig. 5D–E). Furthermore, the WT group showed elevated NADPH concentrations (Fig. 5F) and decreased NADP levels (Fig. 5G). Intermediate glycolytic metabolites, including glucose-6-phosphate, fructose-6-phosphate, fructose-1,6-bisphosphate, glyceraldehyde-3-phosphate, phosphoenolpyruvate, and 2-phosphoglycerate were reduced (Fig. 5H-M), whereas pyruvate levels were elevated (Fig. 5N). Notably, the HNRNPA1-K350R group largely mirrored the metabolic profile of the knockout group, suggesting that lactylation at K350 is essential for driving these metabolic changes.

We next performed functional glycolysis assays. Glucose uptake assays demonstrated that HNRNPA1-WT cells consumed significantly more glucose from the medium than K350R or KO cells (Fig. 5O). Consistently, lactate production (measured as lactate released into the culture medium) was markedly higher in WT cells and was significantly reduced in K350R and KO cells (Fig. 5P). These results align with the metabolomics data, confirming that HNRNPA1 lactylation boosts glycolytic flux.

Measurements of cellular ATP content showed that HNRNPA1-KO and K350R cells had higher ATP levels than WT cells (Fig. 5Q). While at first counterintuitive (one might expect high glycolysis cells to have more ATP), this finding likely reflects the fact that WT cells rely on less efficient ATP production (glycolysis yields fewer ATP per glucose than oxidative phosphorylation). The higher ATP in K350R and KO cells suggests a relative shift towards oxidative phosphorylation (OXPHOS) in the absence of lactylated HNRNPA1. In line with this, we measured the extracellular acidification rate (ECAR), an indicator of glycolytic activity, under basal and stress conditions. Both basal ECAR and maximal glycolytic capacity were significantly elevated in HNRNPA1-WT cells compared to K350R and KO cells (Fig. 5R-S).

We also assessed cellular redox status by examining the NADPH/NADP^+^ ratio and the reduced glutathione/oxidized glutathione (GSH/GSSG) ratio, which reflect the antioxidant capacity of cell. Rapidly proliferating tumor cells often increase NADPH production to cope with elevated reactive oxygen species (ROS). In our experiments, HNRNPA1-WT cells had significantly higher NADPH/NADP^+^ ratios and GSH/GSSG ratios than K350R or KO cells (Fig. 5T-U), indicating that lactylation of HNRNPA1 enhances the antioxidant capacity and reductive biosynthesis potential of BLCA cells. When HNRNPA1 is not lactylated (K350R or absent), cells exhibit lower NADPH and GSH relative to their oxidized forms, potentially rendering them more susceptible to oxidative stress.

Finally, we measured intracellular glucose levels as an inverse indicator of consumption rate. HNRNPA1-WT cells had the lowest steady-state glucose content, whereas K350R and KO cells showed significantly higher intracellular glucose (Fig. 5V). This is consistent with the notion that WT cells are avidly consuming glucose, leaving little unused, while K350R/KO cells consume glucose less vigorously.

In summary, these data show that HNRNPA1-K350 lactylation reprograms BLCA cell metabolism toward enhanced aerobic glycolysis. The metabolic consequences of HNRNPA1-K350 lactylation provide a mechanistic basis for its protumor effects.

### P300 functions as the acyltransferase for HNRNPA1-K350 Lactylation

**Fig. 6:**
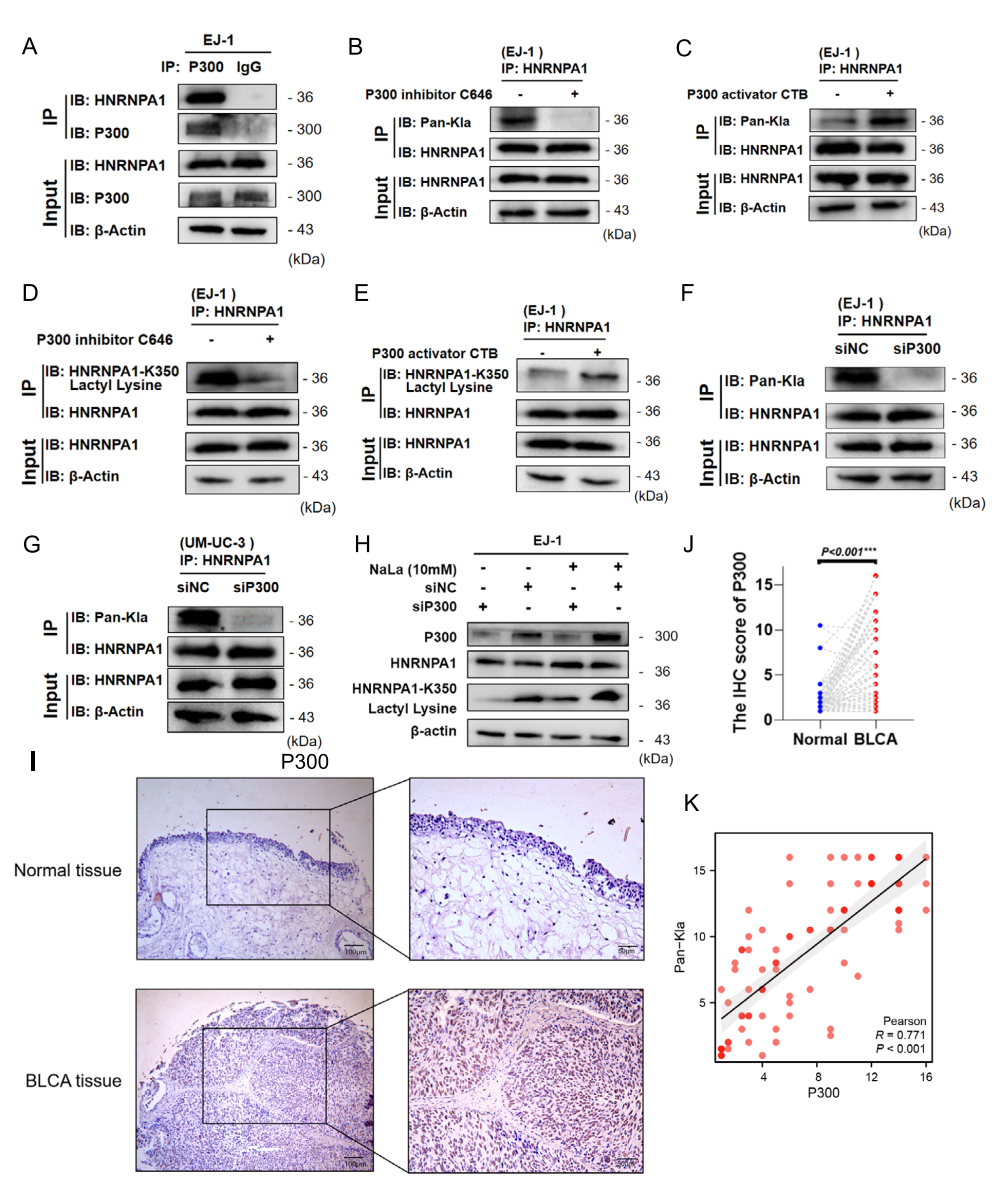
P300 functions as the acyltransferase for HNRNPA1-K350 lactylation. **A** EJ-1 cells were lysed and immunoprecipitated using anti-P300 antibody or control IgG, followed by detection of HNRNPA1. **B-C** EJ-1 cells were lysed and immunoprecipitated using anti- HNRNPA1 antibody, followed by detection of Pan-Kla. **D-E** EJ-1 cells was lysed and immunoprecipitated using anti-HNRNPA1 antibody, followed by detection of HNRNPA1-K350 lactyl lysine. (**F-G** Lactylation levels were detected in EJ-1 and UM-UC-3 cells using immunoprecipitation to detect HNRNPA1 after silencing P300. **H** Western blot analysis of HNRNPA1 K350 lactylation level. HNRNPA1 K350 lactylation level was detected with NaLa treatment or P300 knockdown. **I** P300 levels in normal and tumor tissues were visualized by immunohistochemical (IHC) staining. Scale bar: 100 μm (left panel), 50 μm (right panel). **J** Paired t-test comparison of IHC score between unpaired normal and tumor samples. **K** The correlation between Pan-Kla levels and P300 levels based on IHC scores. Pearson correlation analysis was used. Each dot represents an individual sample. ***p < 0.001

Given the pivotal role of HNRNPA1-K350 lactylation in BLCA progression and metabolic reprogramming, we sought to identify the enzymatic machinery responsible for this PTM. Among several candidate acyltransferases, including P300, CBP, GCN5, and PCAF [8, 23–25], we specifically focused on P300, previously reported to catalyze histone lactylation. Notably, only P300 overexpression significantly elevated HNRNPA1 lactylation levels (Figure S7A). To further investigate the interaction between P300 and HNRNPA1, we performed co-immunoprecipitation (Co-IP) assays in both BLCA and HEK-293 T cells, confirming a direct association (Fig. 6A, Figure S7B). Overexpression of P300 led to increased HNRNPA1 lactylation (Figure S7C–D). However, this effect was absent in cells expressing the HNRNPA1-K350R mutant, indicating the site-specific nature of P300-mediated lactylation (Figure S7E).

We next modulated P300 activity pharmacologically. Treating EJ-1 cells with a P300 catalytic inhibitor significantly reduced HNRNPA1-K350 lactylation levels, whereas treating with a small-molecule P300 activator enhanced HNRNPA1 lactylation (Fig. 6B–E). Similarly, siRNA-mediated knockdown of P300 led to a marked decrease in HNRNPA1 lactylation (Figure S7F, Fig. 6F–G). Western blot analysis showed that P300 knockdown markedly diminished HNRNPA1 lactylation, even after NaLa treatment (Fig. 6H). These results demonstrate that P300 is required for lactate-induced HNRNPA1 lactylation, confirming its role as a key lactyltransferase mediating this modification.

If P300-mediated HNRNPA1 lactylation is functionally important for BLCA, then P300 itself should influence BLCA cell malignant phenotypes. Indeed, P300 knockdown in EJ-1 and UM-UC-3 cells resulted in significantly reduced cell proliferation (Figure S7G). Migration and invasion assays showed that P300-silenced cells had impaired motility and invasiveness compared to control cells (Figure S7H-J). These phenotypic effects mirror those observed when HNRNPA1 lactylation was blocked, reinforcing the connection that P300’s protumor effects are likely mediated via HNRNPA1 lactylation.

We also examined P300 expression in human samples. IHC staining on BLCA patient tissue microarrays revealed that P300 levels were consistently higher in tumor tissues than in adjacent normal tissues (Fig. 6I-J), aligning with a role for P300 in supporting BLCA progression. The correlation analysis shows that level of Pan-Kla is highly positively correlated with the expression of P300 (r = 0.771, *p* < 0.001, Fig. 6K). Together, our findings identify P300 as the key acyltransferase responsible for HNRNPA1-K350 lactylation in BLCA. Given P300’s broad role in adding acyl groups to histones and other proteins, it likely serves as the “writer” that attaches lactate to HNRNPA1, thereby activating the downstream effects on splicing and metabolism.

### HNRNPA1-K350 lactylation drives PKM2 splicing to promote aerobic glycolysis and tumor aggressiveness

**Fig. 7:**
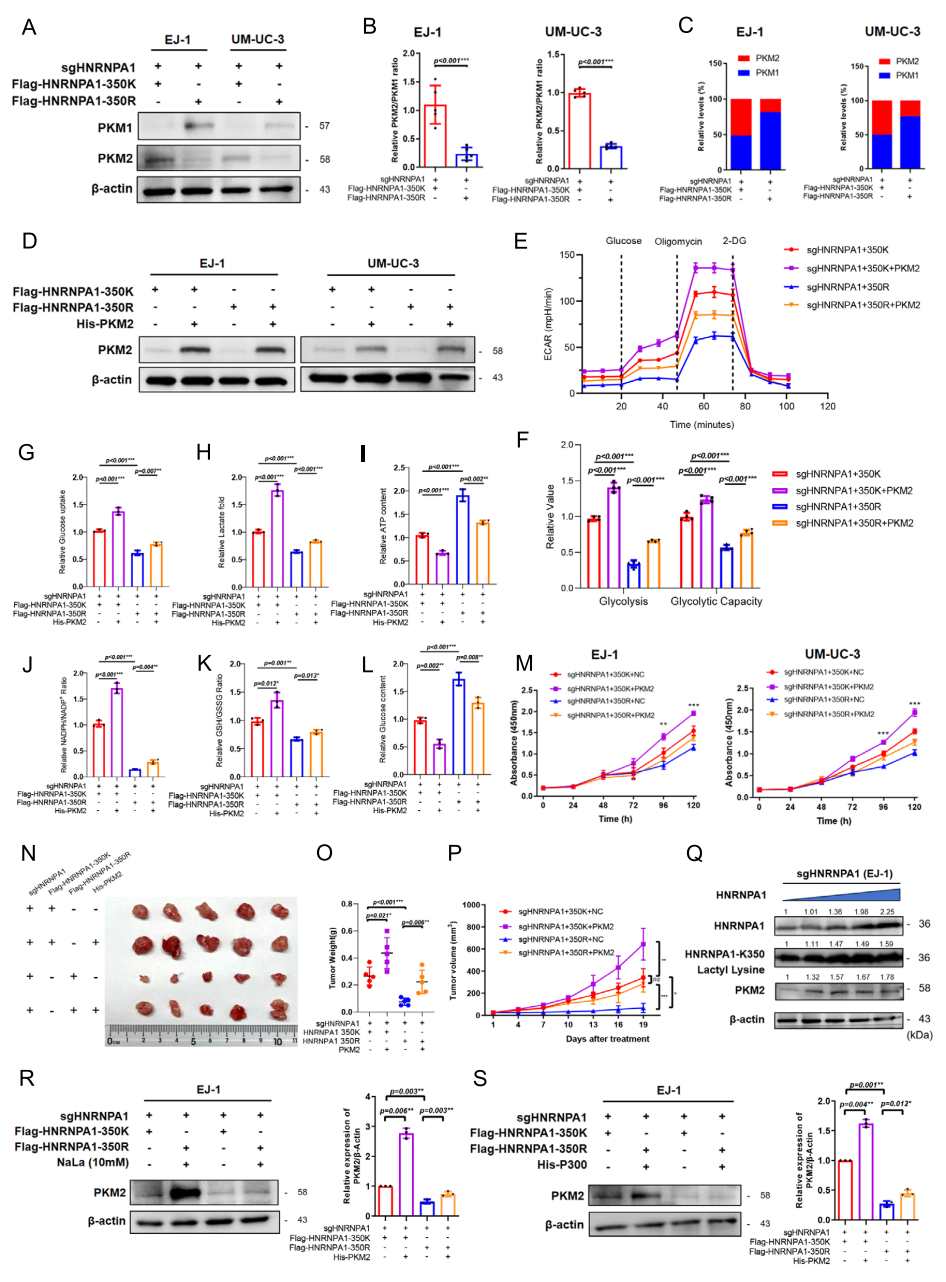
HNRNPA1-K350 lactylation mediates aerobic glycolysis and promotes the proliferation of bladder cancer cells via PKM splicing. **A** Western blot was used to detect the expression levels of PKM1 and PKM2 in bladder cancer cells (**B-C**) Changes in the ratio of PKM2 to PKM1 and the relative expression levels of PKM2 and PKM1 were detected by qPCR. **D** Western blot was used to detect the overexpression efficiency of PKM2 in BLCA cells. (E–F) Extracellular acidification rate assay to detect basal glycolysis and glycolytic capacity. **G-H** Glucose uptake and lactate production were detected. **I** ATP content was determined by ATP assay kit. **J** NADPH/NADP^+^ assay to detect alterations of NADPH/NADP.^+^ ratio. **K** GSH/GSSG assay to detect GSH/GSSG ratio. **L** Glucose content assay to determine the relative intracellular glucose content. (M) Proliferation of bladder cancer cells was analyzed using CCK-8 assay. **N–O** Nude mice were injected with EJ-1 cells subcutaneously. Tumors were harvested on Day 19 after subcutaneous implantation. Tumor gross image and tumor weight after sacrifice. Statistical analyses were performed with one-way ANOVA (*n* = 5). **P** Tumor volume assessment during the experiment. **Q-S** Western blot analysis of PKM2 level. PKM2 level was detected with different amount of HNRNPA1 (**Q**), NaLa (**R**) or P300 knockdown (**S**). All data are presented as mean ± SD. *p < 0.05, **p < 0.01, ***p < 0.001

In mammals, aerobic glycolysis is partly regulated by pyruvate kinase isoform expression. Cancer cells predominantly express the embryonic isoform PKM2, which promotes aerobic glycolysis, while the adult isoform PKM1 supports OXPHO [26]. These isoforms arise from alternative splicing of a shared pre-mRNA, governed by HNRNPs, which favor exon 10 inclusion to drive PKM2 expression [27]. Although PKM2 upregulation has been reported in BLCA [28], the role of HNRNPA1, an established splicing regulator, remains unclear, particularly in relation to its lactylation. We therefore investigated whether HNRNPA1-K350 lactylation modulates PKM splicing and contributes to BLCA progression. Western blot and qPCR analyses revealed that overexpression of HNRNPA1-WT led to increased PKM2 protein and mRNA levels, accompanied by a reduction in PKM1 expression. In contrast, expression of the HNRNPA1-350R mutant favored PKM1 expression over PKM2 (Fig. 7A–B). Furthermore, the proportion of PKM2 was significantly higher in cells expressing HNRNPA1-WT (Fig. 7C), suggesting that HNRNPA1-K350 lactylation skews PKM splicing toward the PKM2 isoform.

To directly link this splicing change to metabolic function, we examined whether the effects of HNRNPA1 lactylation on glycolysis could be rescued by manipulating PKM2 levels. We ectopically overexpressed PKM2 in HNRNPA1-K350R cells to see if it could restore their glycolytic capacity (Fig. 7D). ECAR measurements showed that HNRNPA1-K350R cells have reduced glycolysis and glycolytic capacity, but when PKM2 was overexpressed in these cells, their ECAR increased to levels similar to WT cells (Fig. 7E). This rescue experiment implies that the Warburg defect caused by loss of HNRNPA1 lactylation can be largely attributed to decreased PKM2.

Similarly, the diminished glucose uptake and lactate secretion observed in HNRNPA1-K350R cells were reversed by PKM2 overexpression (Fig. 7G-H). The elevated ATP levels (indicative of reliance on OXPHOS) in K350R cells dropped back down upon PKM2 reintroduction (Fig. 7I), consistent with a shift back toward glycolysis. Furthermore, the compromised NADPH/NADP^+^ and GSH/GSSG ratios in K350R cells were restored when PKM2 was replenished (Fig. 7J-K). Intracellular glucose accumulation in K350R cells (due to slower glycolysis) was also alleviated by PKM2 overexpression, as more glucose was metabolized (Fig. 7L). Collectively, these metabolic rescues confirm that PKM2 is a critical downstream effector of HNRNPA1 lactylation’s impact on cellular metabolism.

To further validate the role of PKM2 in mediating the oncogenic effects of HNRNPA1-K350 lactylation, we performed functional assays both in vitro and in vivo. In HNRNPA1-K350R cells, forced expression of PKM2 significantly increased cell proliferation (Fig. 7M) and partially restored tumor growth (Fig. 7N–P). In wound-healing and invasion assays, PKM2 overexpression counteracted the reduced migration/invasion phenotype of K350R cells, as evidenced by increased wound closure and more cells invading through Matrigel (Figure S8A–F). These findings tie the pro-malignant effects of HNRNPA1 lactylation to its promotion of PKM2-driven metabolism.

To further examine whether HNRNPA1 exerts dose-dependent effects on PKM2 regulation, we transfected sgHNRNPA1 EJ-1 cells with increasing doses of an HNRNPA1 overexpression plasmid. As the expression of HNRNPA1 gradually increased, a corresponding elevation in HNRNPA1-350Kla was observed. Notably, the expression of PKM2 also rose in parallel with HNRNPA1 levels, indicating that PKM2 expression is both HNRNPA1 dose-dependent and lactylation-dependent. These findings suggest that HNRNPA1 promotes glycolytic activation in a manner tightly coupled to its own lactylation status (Fig. 7Q). We also found that treating BLCA cells with NaLa or overexpressing P300 led to upregulation of PKM2 protein in HNRNPA1-WT cells, but not in K350R cells (Fig. 7R-S). Conversely, inhibiting glycolysis (with 2-DG or oxamate) or knocking down LDHA reduced PKM2 expression in cells, consistent with the idea that glycolysis-derived lactate is needed to maintain PKM2 via HNRNPA1 lactylation (Figure S9G–I).

Collectively, these results indicate that HNRNPA1-K350 lactylation regulates alternative splicing of PKM to favor the PKM2 isoform, thereby promoting the aerobic glycolysis and supporting the aggressive phenotype of BLCA cells.

### Small molecule drug inhibits the lactylation and biological activity of HNRNPA1 lactylation in bladder cancer

**Fig. 8:**
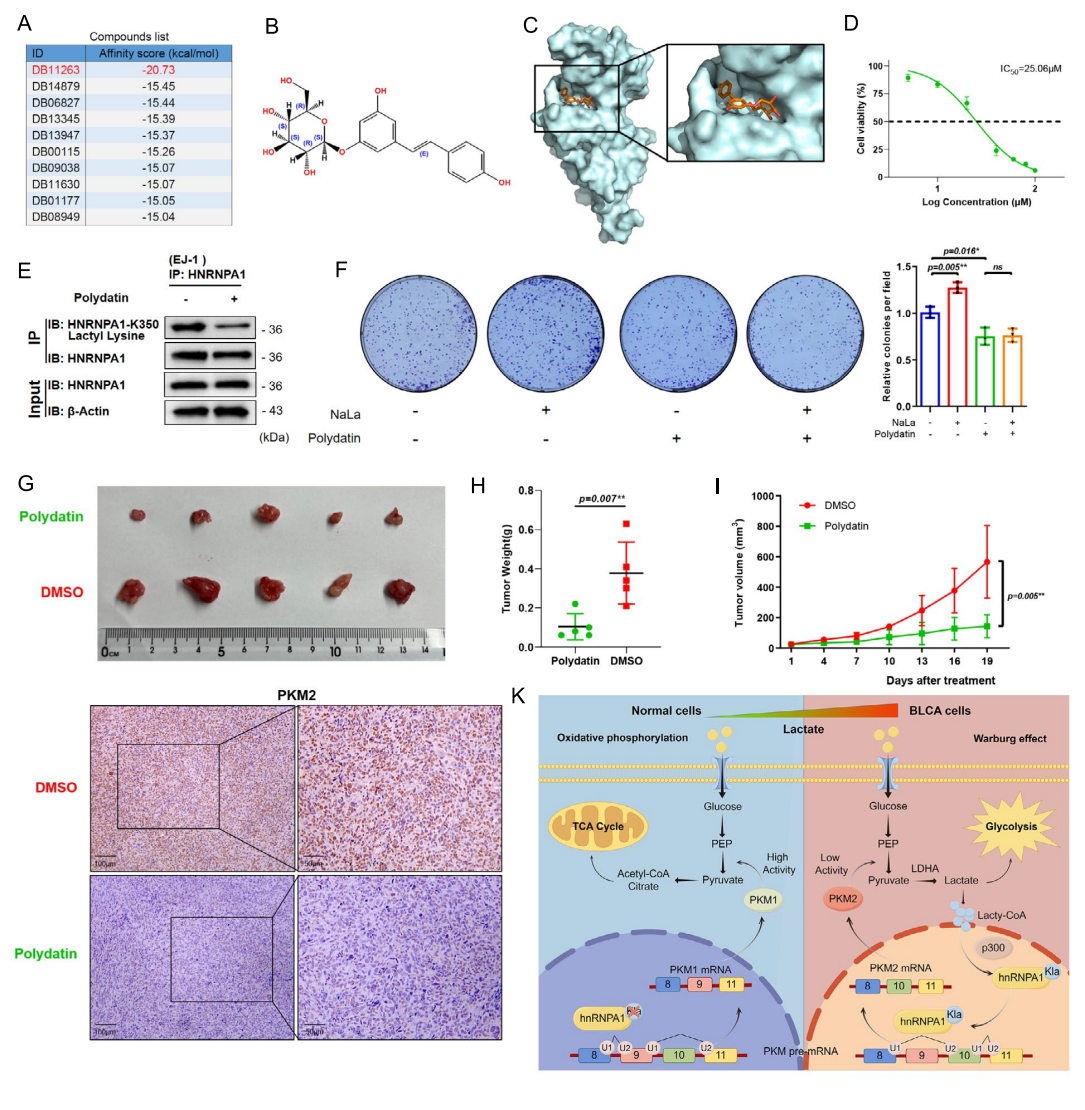
Polydatin inhibits HNRNPA1 lactylation and its biological activity in bladder cancer. **A** Binding free energy of ten candidate compounds docked to the HNRNPA1 active pocket. **B** Chemical structure of polydatin. **C** Docking visualization of polydatin within the active pocket of HNRNPA1. **D** IC_50_ values of polydatin in the EJ-1 cell line, as determined by CCK8 assay. **E** EJ-1 cells were lysed and immunoprecipitated using an anti-HNRNPA1 antibody, followed by detection of HNRNPA1-K350 lactyl lysine. **F** Colony formation assays were performed to assess the proliferative capacity of EJ-1 cells following 24-h treatment with 30 μM polydatin or 20 mM NaLa. **G-H** Tumor gross images and tumor weights were assessed in nude mice after subcutaneous injection of EJ-1 cells. Tumors were harvested on Day 19 after subcutaneous implantation. Statistical analyses were performed with unpaired two-tailed Student’s t-test (*n* = 5). **I** Tumor volume measurements throughout the experiment. **J** Immunohistochemical staining visualized PKM2 levels in xenograft tumors. Scale bars: 100 μm (left panel), 50 μm (right panel). **K** Schematic representation of the proposed mechanism of HNRNPA1 lactylation. *p < 0.05, **p < 0.01

Given that HNRNPA1 lactylation drives PKM2 splicing and promotes BLCA progression, it may represent a promising therapeutic target. Subsequently, we further explored the potential druggability of the binding pocket of HNRNPA1. We identified ten candidates with relatively low binding energies (Fig. 8A), among which polydatin had the lowest binding energy. The molecular formula of polydatin is shown in Fig. 8B, and it interacts effectively with the amino acid residues near the active site pocket of HNRNPA1 (Fig. 8C). Furthermore, we investigated the inhibitory effect of polydatin on cell proliferation. And we found that. increasing the abundance of polydatin in the culture medium results in a concentration-dependent inhibition of cell proliferation, with an IC_50_ value of 25.06 μM (Fig. 8D). Meanwhile, we noted that polydatin can block the lactylation of HNRNPA1 K350. Under conditions of exogenous lactate addition, polydatin also inhibited cell colony formation of EJ-1 cells (Fig. 8F). Polydatin also significantly inhibited tumor proliferation in vivo (Fig. 8G-I) and and the PKM2 staining also indicate that polydatin can block variable shear event of PKM2 caused by HNRNPA1 lactylation (Fig. 8J), demonstrating its antitumor efficacy in BLCA. These data confirm that polydatin is a potential drug targeted at HNRNPA1 in BLCA.

## Discussion

Metabolic reprogramming has long been recognized as a hallmark of cancer, providing tumor cells with the energy and building blocks required for rapid growth [29]. Among the metabolic alterations, aerobic glycolysis (the Warburg effect) is prominent in many cancers, including bladder cancer. Warburg metabolism leads to excessive lactate production, which has traditionally been viewed as a metabolic waste product. However, emerging evidence indicates that lactate plays active roles in cancer biology beyond serving as a fuel for oxidative metabolism in oxygenated cells. Lactate can acidify the tumor microenvironment, promoting invasion and immune evasion, and can act as a signaling molecule and substrate for epigenetic modifications [30]. Our study adds to this evolving understanding by demonstrating that lactate drives protein lactylation to rewire gene regulation in BLCA.

High lactylation levels in BLCA tissues were strongly associated with unfavorable clinicopathological features (high grade, advanced stage) and shorter patient survival, suggesting that lactylation might serve as a biomarker of aggressive disease. This is in line with reports in other cancers where global lactylation or specific lactylation events correlate with malignancy [31]. Notably, our data show that lactylation is not merely a correlate but a driver of oncogenic behavior in BLCA. When we inhibited lactate production or lactylation, tumor cell proliferation and invasion were significantly impaired. Conversely, increasing lactylation enhanced tumor cell growth, even in non-cancerous cells. Notably, both 10 mM and 20 mM NaLa treatments markedly increased global protein lactylation, yet resulted in comparable proliferative effects in SV-HUC-1 cells. Although lactate-induced lactylation promotes cell proliferation, its stimulatory effect appears to be limited in non-tumorigenic cells. This plateau phenomenon likely reflects a functional saturation of the lactylation-mediated signaling cascade, in which moderate lactate exposure is sufficient to activate P300 and downstream pathways, whereas further elevation of extracellular lactate fails to enhance proliferative output due to transporter saturation and cellular metabolic feedback regulation.

Through an unbiased proteomic approach, we identified HNRNPA1 as a key lactylation target in BLCA. HNRNPA1 is a well-characterized RNA-binding protein that influences alternative splicing and various aspects of mRNA processing. Intriguingly, our results indicate that the oncogenic activity of HNRNPA1 in BLCA is not solely a function of its expression level, but critically depends on its PTM by lactylation at lysine 350. This exemplifies a metabolic regulation paradigm wherein the cellular metabolic state, indicated by lactate levels, directly modulates HNRNPA1’s splicing regulatory function.

In the context of cancer metabolism, one of the most consequential splicing decisions is the production of PKM2 versus PKM1 isoforms from the PKM gene [32]. PKM2 is prevalent in embryonic and cancerous tissues and facilitates aerobic glycolysis, whereas PKM1 is predominant in normal differentiated tissues and promotes oxidative metabolism. It has been established that HNRNPs are splicing repressors of PKM exon 9, thereby favoring PKM2 generation [27]. Our study extends this knowledge by showing that HNRNPA1’s ability to promote PKM2 splicing in BLCA is enhanced by its lactylation. When HNRNPA1 cannot be lactylated, the balance of PKM splicing shifts, resulting in more PKM1 and less PKM2, which in turn dampens glycolytic flux and tumor growth. This establishes a mechanistic link between the high lactate tumor microenvironment and the persistence of the Warburg effect, where lactate enhances the expression of PKM2, the enzyme that promotes further lactate production, forming a self-sustaining feedforward loop (Fig. 8K).

P300 is recognized as one of the most versatile and complex acyltransferases involved in both physiological and pathological processes. It functions as a writer protein for a wide range of acyl modifications, including acetylation, succinylation, and β-hydroxybutyrylation [24, 33, 34]. Our findings suggest that P300 can utilize lactyl-CoA (derived from lactate) to lactylate specific protein targets. In our case, P300 was necessary and sufficient to lactylate HNRNPA1 at K350. Targeting P300 in cancer has been explored mainly to block its acetyltransferase activity on histones and oncogenic transcription factors [24]. Our study provides another rationale, as P300 inhibition could simultaneously blunt lactylation-driven cancer-promoting pathways.

While the initial reports of lactylation were focused on histone modifications in macrophages [8], it is increasingly clear that lactylation extends to non-histone proteins and can impact diverse cellular pathways. For instance, lactylation has been found to regulate the immune response in sepsis by modifying key inflammatory regulators [35], and to influence cardiac fibrosis in heart failure models through modification of cardiac proteins [36]. In cancer, emerging studies have identified lactylation of metabolic enzymes, chromatin modifiers, and even oncoproteins, suggesting this PTM might be widespread [37, 38]. Our identification of HNRNPA1 adds an RNA-binding protein to the list and underscores that lactylation can directly affect RNA splicing, a new dimension of metabolic control over the transcriptome.

From a therapeutic perspective, we conducted virtual screening within an approved drug library to identify active compounds capable of blocking HNRNPA1 function, and found that polydatin exhibited the lowest binding energy. Polydatin is a naturally occurring polyphenolic compound derived from the root of *Polygonum cuspidatum*, widely recognized for its anti-inflammatory, antioxidant, and anticancer properties. It is a glycoside of resveratrol, and upon hydrolysis, it releases resveratrol, which is known for its potent biological activities. In the context of cancer, polydatin has shown promise in various tumor types, where it exerts anti-proliferative effects by inducing apoptosis, inhibiting cell migration, and modulating key signaling pathways involved in cancer progression [39, 40]. In this study, we found that polydatin can reduce the lactylation of HNRNPA1, thereby blocking its biological effects and simultaneously decreasing PKM2 expression. Therefore, polydatin emerges as a promising candidate for targeting the HNRNPA1-K350 lactylation/PKM2 axis. However, the exact underlying mechanisms require further investigation to fully understand its therapeutic potential.

Although the present study primarily focused on the cell-intrinsic effects of lactylation within cancer cells, particularly the HNRNPA1–PKM2 regulatory axis, it is important to note that lactylation may also play a pivotal role in shaping the tumor microenvironment (TME). Accumulating evidence indicates that lactate-derived protein lactylation can modulate immune cell function, such as promoting M2 macrophage polarization, suppressing cytotoxic T-cell activation, and altering cytokine secretion patterns, thereby facilitating tumor progression and immune evasion [30, 41, 42]. Given that the current in vivo experiments were performed in nude mice with a subcutaneous xenograft model, the contribution of the immune microenvironment could not be fully evaluated. Future studies using immune-competent mouse models or tumor–immune co-culture systems will be necessary to determine how lactylation and its inhibition influence the interplay between BLCA cells and immune components within the TME. Such investigations will further elucidate the systemic role of lactylation in tumor metabolism and immunoregulation.

In summary, our study offers novel insights and significant contributions to the understanding of epigenetic regulation in the metabolic reprogramming of BLCA. We demonstrate that lactylation levels are inversely correlated with tumor stage, histological grade, and patient prognosis, suggesting its potential as a prognostic biomarker. Mechanistically, we uncover that lactate accumulation within the TME facilitates abnormal glycolysis via HNRNPA1-K350 lactylation, which in turn regulates the alternative splicing of PKM to favor the oncogenic PKM2 isoform. A small-molecule inhibitor attenuates cellular proliferation by binding to the active pocket of HNRNPA1, thereby suppressing the expression of PKM2. This process establishes a glycolysis–HNRNPA1-K350 lactylation–PKM2 positive feedback loop that drives BLCA initiation and progression. Collectively, our findings highlight this regulatory axis as a promising target for the development of complementary therapeutic strategies in the clinical management of BLCA.

## Supplementary Information

Supplementary Material.

## Data Availability

The data that support the findings of this study are available from the corresponding author upon reasonable request.
