# Supplementary material for: Lactate-driven lactylation of HNRNPA1 orchestrates PKM2 splicing and glycolytic reprogramming in bladder cancer
Source: J Exp Clin Cancer Res. 2025 Nov 22;44:331. doi: 10.1186/s13046-025-03591-5 (PMC12752355; doi:10.1186/s13046-025-03591-5)
Supplement: Supplementary file 1 — Supplementary Material. [file 13046_2025_3591_MOESM1_ESM.pdf]

Supplementary Materials for:

Lactate-Driven Lactylation of HNRNPA1 Orchestrates PKM2 Splicing and Glycolytic Reprogramming in Bladder Cancer

Tianqi Wang, Xiaohong Ma, Yini Wang, Hongquan Liu, Guixin Ding, Yanfei Li, Hejia Yuan, Jie Gao, Fengze Sun, Yicheng Guo, Jian Ma and Jitao Wu

CONTENTS

**Supplementary Figures**		**Page**
Materials and methods		2
Supplementary Figure 1.	Gene Set Enrichment Analysis (GESA) revealed significant enrichment of Glycolysis.	5
Supplementary Figure 2.	Suppressive effect of glycolysis inhibitors or LDHA silencing on the malignant phenotype of bladder cancer cells.	6
Supplementary Figure 3.	Quality control of lactylation modification in bladder cancer and adjacent normal tissues.	8
Supplementary Figure 4.	Enrichment analysis of lactylation modification in bladder cancer and adjacent normal tissues.	9
Supplementary Figure 5.	HNRNPA1 K350 is the lactylation site of HNRNPA1 in bladder cancer.	10
Supplementary Figure 6.	Suppressive effect of HNRNPA1 knockout on the malignant phenotype of bladder cancer cells.	11
Supplementary Figure 7.	P300 is the acyltransferase of HNRNPA1 K350 lactylation.	12
Supplementary Figure 8.	HNRNPA1-K350 lactylation sustains aggressive phenotype via PKM2 splicing.	14
**Supplementary Tables**		
Supplementary Table 1.	Clinical characteristics data	16
Supplementary Table 2.	Plasmids and siRNA used in this study	19
Supplementary Table 3.	Reagents used in this study	20
Supplementary Table 4.	Antibody used in this study	21
Supplementary Table 5.	Primer used in this study	22
Supplementary Table 6.	Lactylation-related genes used for single-cell analysis	23

Materials and methods

Trypsin Digestion: Equal amounts of total protein from each sample were subjected to enzymatic digestion to ensure consistent sample volumes. Trichloroacetic acid (TCA) was slowly added to the protein solution to reach a final concentration of 20%, followed by vortex mixing and incubation at 4 °C for 2 hours to precipitate proteins. The samples were centrifuged at 4,500×g for 5 minutes, and the resulting precipitate was washed three times with pre-chilled acetone. After drying, the precipitate was resuspended in 200 mM triethylammonium bicarbonate (TEAB) buffer with sonication. Trypsin was added at a 1:50 (enzyme:protein, w/w) ratio for overnight digestion at 37 °C. Subsequently, dithiothreitol was added to a final concentration of 5 mM, and the samples were incubated at 37 °C for 1 hour. Iodoacetamide was then introduced at 11 mM, followed by incubation for 45 minutes at room temperature in the dark.

Enrichment of PTM Peptides: The resulting peptides were resuspended in immunoprecipitation (IP) buffer containing 100 mM NaCl, 1 mM EDTA, 50 mM Tris-HCl, and 0.5% NP-40 (pH 8.0). The peptide solution was incubated with pre-washed anti-lysine lactylation remnant antibody resin (PTM-1404, PTM Biolabs, China) at 4 °C overnight with gentle rotation. Following incubation, the resin was washed thoroughly with IP buffer and deionized water. Lactylated peptides were eluted three times using 0.1% trifluoroacetic acid (TFA) and subsequently purified using C18 ZipTips (Millipore, USA) prior to mass spectrometry analysis.

LC-MS/MS analysis: For lactylation-specific analysis, tryptic peptides were reconstituted in mobile phase A and subjected to chromatographic separation using a NanoElute ultra-high-performance liquid chromatography (UHPLC) system (Bruker Daltonics, Germany). Mobile phase A consisted of 0.1% formic acid and 2% acetonitrile in water, while mobile phase B comprised 0.1% formic acid in acetonitrile. Peptides were separated on a reversed-phase analytical column (25 cm length, 100 µm inner diameter, 1.9 µm particle size) using the following elution gradient: 0–72 min,

7%–24% B; 72–84 min, 24%–32% B; 84–87 min, 32%–80% B; and 87–90 min, 80% B. The flow rate was maintained at 450 nL/min throughout the run. Mass spectrometry analysis was conducted using a timsTOF Pro mass spectrometer (Bruker Daltonics) equipped with a nano-electrospray ion source operating at 1.7 kV. The instrument was operated in Parallel Accumulation Serial Fragmentation (PASEF) mode, with data acquisition over an m/z range of 100–1700. Precursors with charge states from 0 to 5 were selected for fragmentation, and 10 PASEF MS/MS scans were acquired per cycle. A dynamic exclusion time of 30 seconds was applied to avoid repeated fragmentation of the same precursor ions. For lactylation-specific analysis, the same NanoElute system and column were used, with the following adjusted elution gradient: 0–44 min, 6%–22% B; 44–54 min, 22%–30% B; 54–57 min, 30%–80% B; and 58–60 min, 80% B. The flow rate and capillary column parameters remained unchanged. Peptides were again analyzed using the timsTOF Pro in PASEF mode under identical conditions, with precursor charge states of 0–5 and 10 PASEF MS/MS scans per cycle. A dynamic exclusion time of 24 seconds was set to minimize redundancy in parent ion selection.

For metabolomics, the Shimadzu Nexera X2 LC-30AD ultra-high-performance liquid chromatography (UHPLC) system, equipped with an XBridge BEH C18 column, was utilized for chromatographic separation. The column temperature was set to 40°C, and the mobile phases consisted of: Phase A, a water solution with 5% acetonitrile and 10 mM ammonium acetate (pH 9.0); and Phase B, a water solution containing 95% acetonitrile and 10 mM ammonium acetate (pH 9.0). The gradient elution program was as follows: 0-2 minutes, 95% Phase B; 2-9 minutes, linearly decrease to 70% Phase B; 9-10 minutes, decrease to 30% Phase B; 10-11 minutes, maintain 30% Phase B; 11-11.5 minutes, increase to 95% Phase B; 11.5-15 minutes, equilibrate to initial conditions. The flow rate was set at 300 μL/min, with an injection volume of 5 μL, and the sample tray was maintained at 4°C. Mass spectrometric analysis was performed using a triple quadrupole linear ion trap mass spectrometer in Multi-reaction Monitoring (MRM) mode, optimized for specific detection based on parent ion-product ion pairs of target metabolites. Collision energy and declustering potential were optimized through

preliminary experiments. Data acquisition and analysis were conducted using Analyst TF 1.7.1 software (AB Sciex), with peak area integration using the Macleod algorithm, and a signal-to-noise (S/N) threshold of ≥3:1.

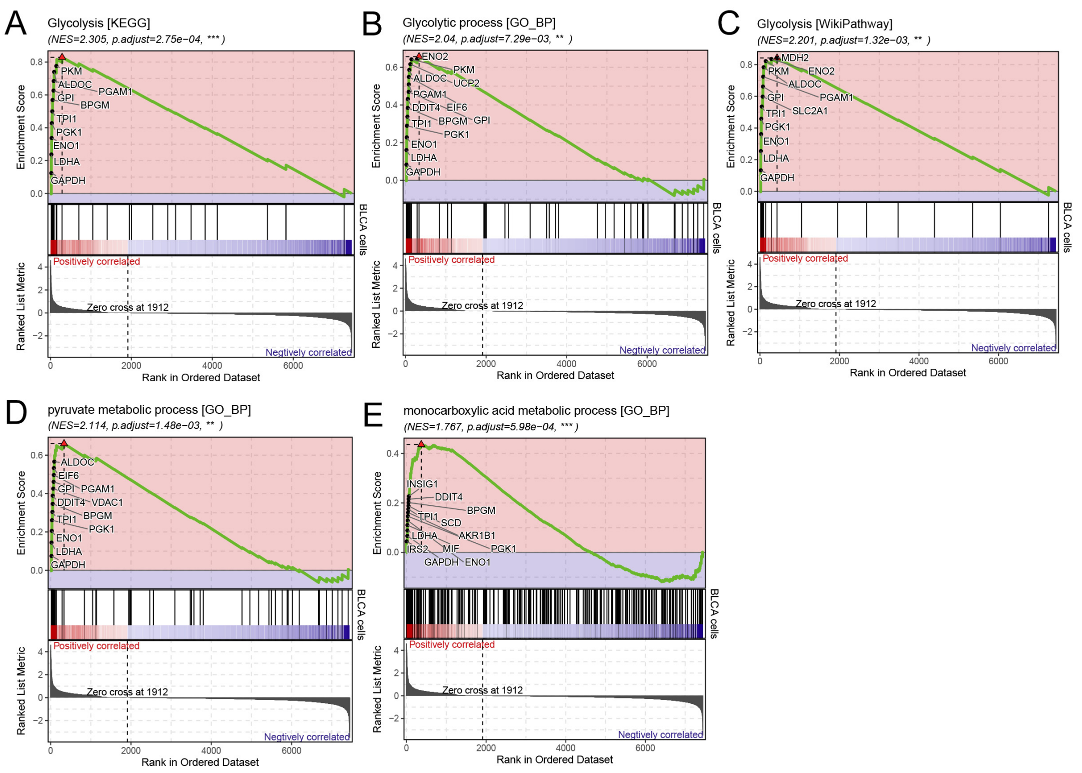

Supplementary Figure 1. Gene Set Enrichment Analysis (GSEA) revealed significant enrichment of Glycolysis. (A, C), Glycolytic Process (B), Pyruvate (D) and monocarboxylic acid metabolic process (E) in bladder cancer cells. $**p < 0.01$, $***p < 0.001$.

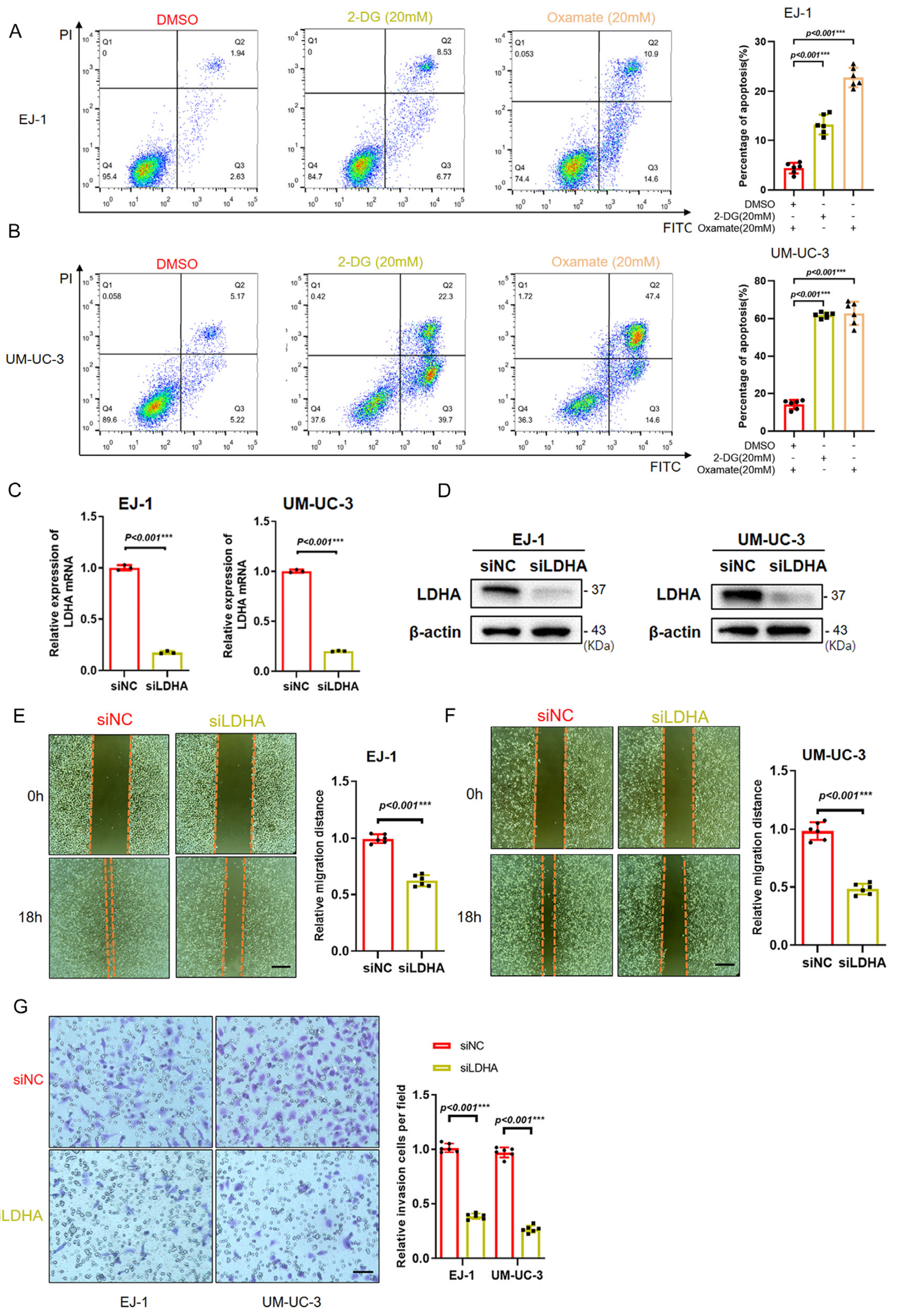

Supplementary Figure 2. Suppressive effect of glycolysis inhibitors or LDHA silencing on the malignant phenotype of bladder cancer cells. (A-B) Cell apoptosis was determined by using flow cytometry. (C) qPCR analysis of LDHA knockdown in EJ-1 and UM-UC-3 cells. (D) Western blots

confirming LDHA knockdown. (E-F) Migration of EJ-1 and UM-UC-3 cells in scratch assays, scale bar: 200 μm. (G) Invasion of EJ-1 and UM-UC-3 cells determined by the Transwell invasion assay, scale bar: 20 μm. ***$p < 0.001$.

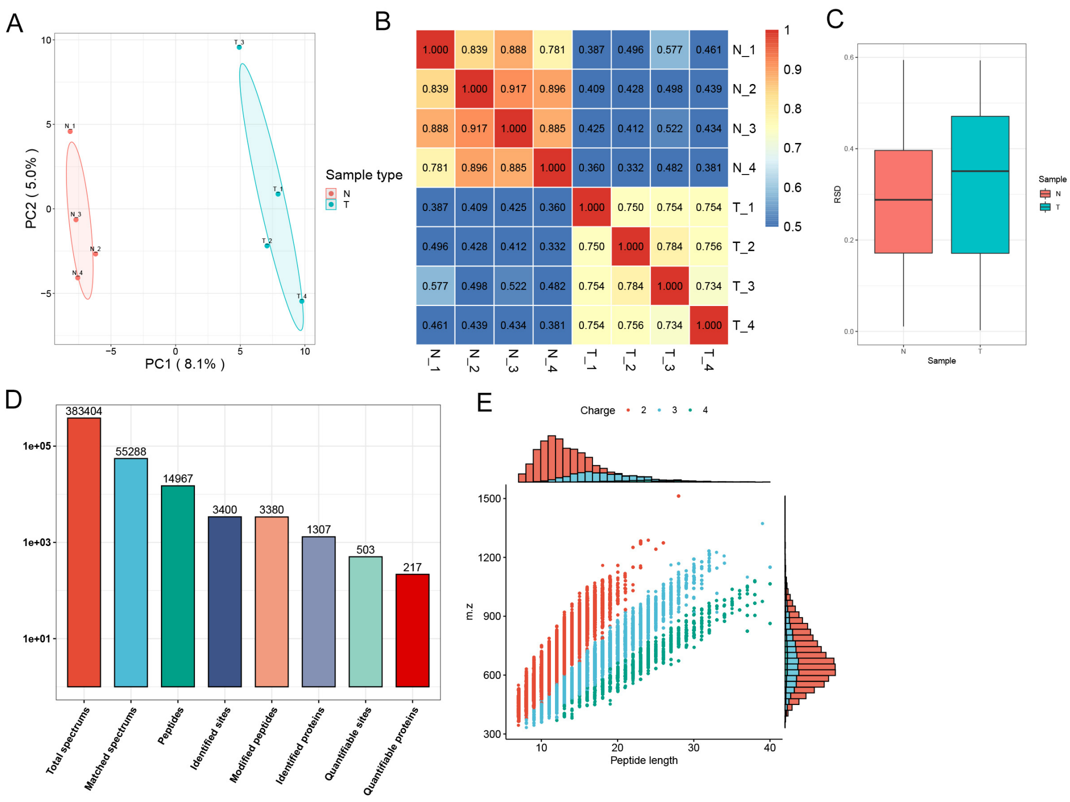

Supplementary Figure 3. Quality control of lactylation modification in bladder cancer and adjacent normal tissues. (A) Principal component analysis (PCA) plot showing the first two principal components of protein intensities; samples are grouped by centroids based on specimen type. (B) Pearson correlation matrix depicting pairwise correlation coefficients between samples. (C) Boxplot of relative standard deviation (RSD) values, with each dot representing an individual protein's RSD. (D) Histogram summarizing the distribution of peptide identifications from mass spectrometry. (E) Length distribution of identified peptides.

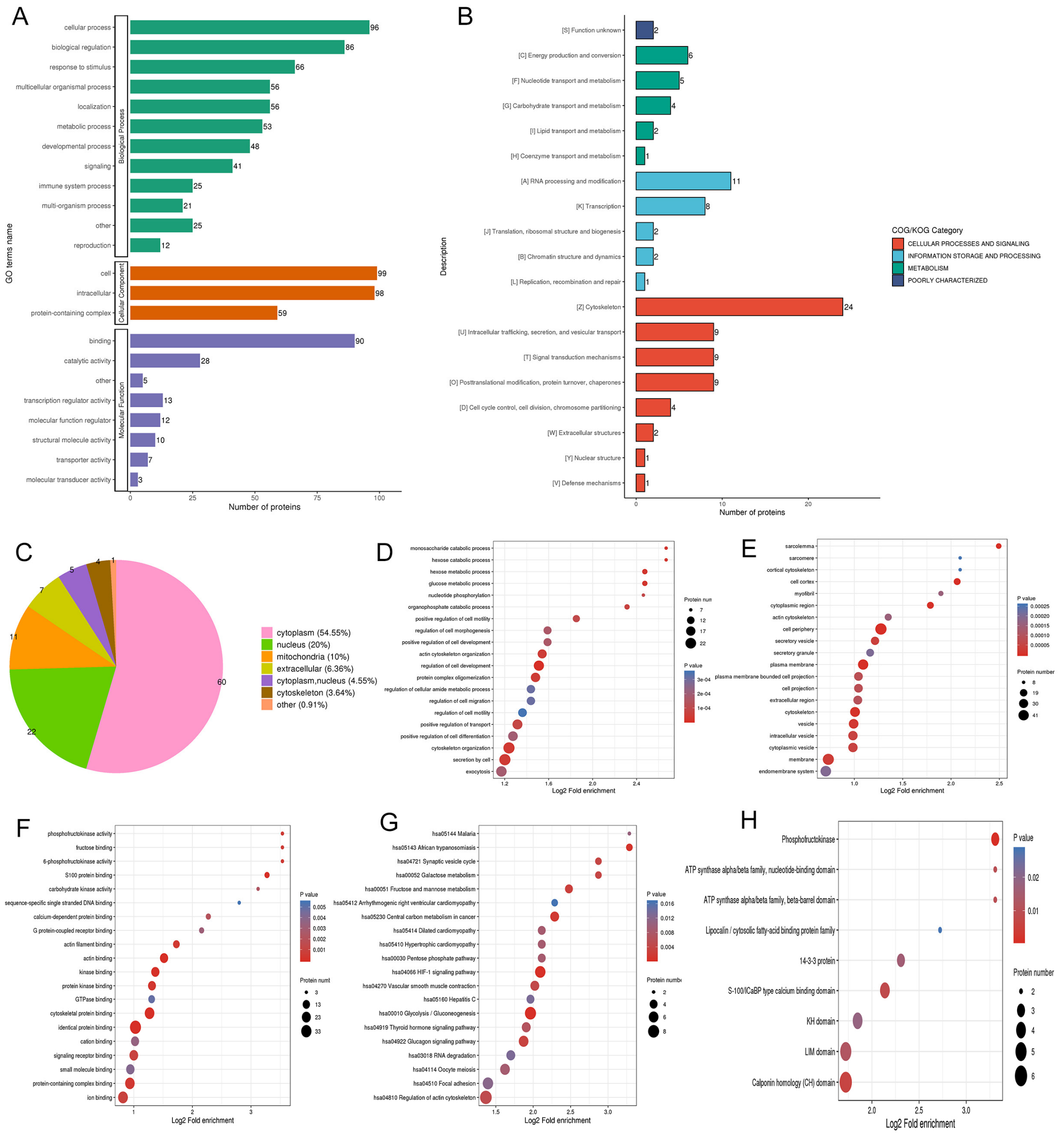

Supplementary Figure 4. Enrichment analysis of lactylation modification in bladder cancer and adjacent normal tissues. (A-C) Differentially lactylation modification proteins were functionally classified according to Gene Ontology (GO) (A), Clusters of Orthologous Groups of proteins (B) and distribution patterns of the subcellular structures (C). (D-H) Analysis of biological process (BP) (D), cellular Component (CC) (E), molecular function (MF) (F), KEGG pathway (G) and protein domain enrichment analysis (H) of differentially lactylation modification proteins, the bubble diagram generated using the ggplot2 tool of the R software showing results.

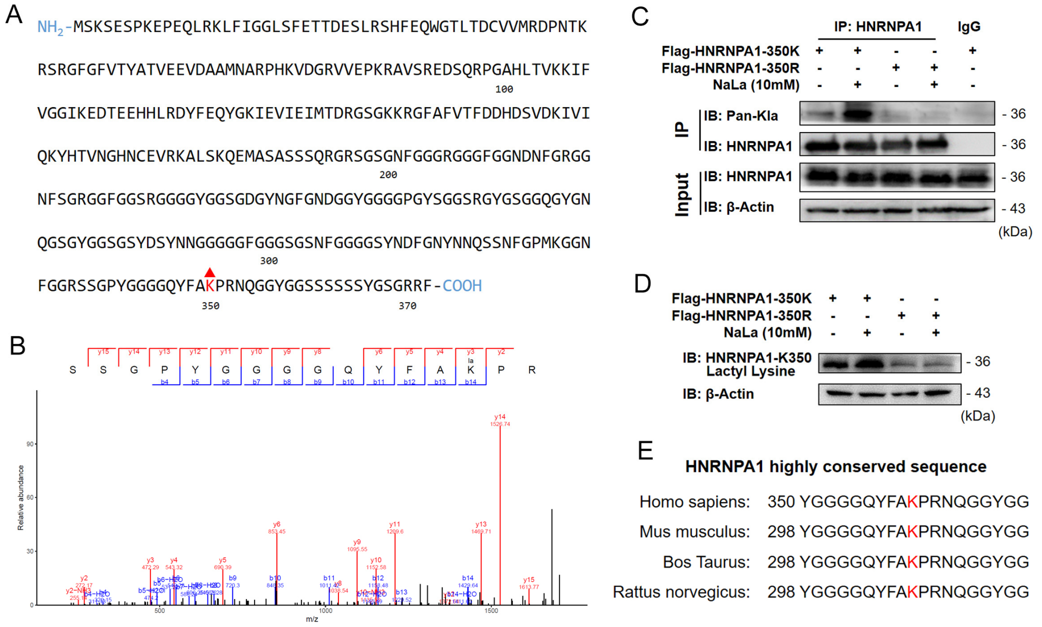

Supplementary Figure 5. HNRNPA1 K350 is the lactylation site of HNRNPA1 in bladder cancer. (A) Mass spectroscopy revealed potential lactylation sites of HNRNPA1. (B) Lactylation site of HNRNPA1 identified by mass spectrometry: K350. (C) Lactylation of K350 site on HNRNPA1 was determined by immunoprecipitation analysis. (D) Western blot analysis to assess HNRNPA1-K350 lactyl lysine level. (E) Lactylated lysine residues were highlighted (red) in alignment of sequences surrounding K350 in HNRNPA1 homologs from diverse species.

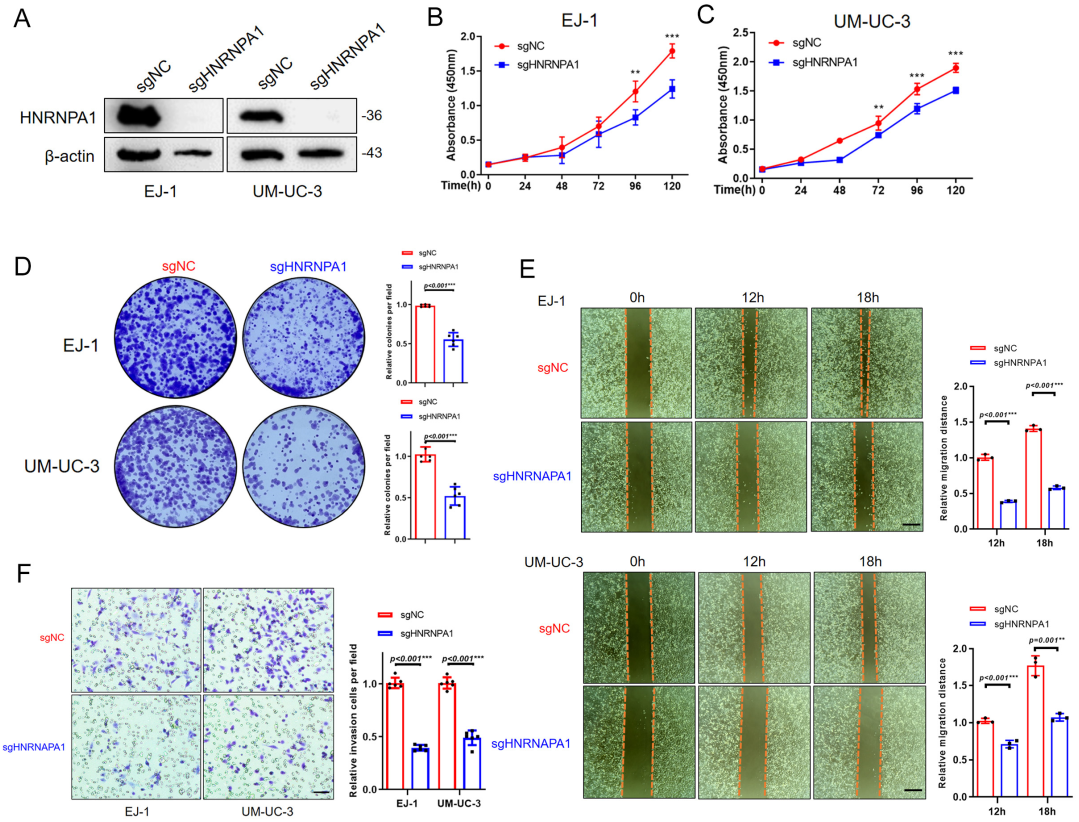

Supplementary Figure 6. Suppressive effect of HNRNPA1 knockout on the malignant phenotype of bladder cancer cells. (A) Western blot analysis to assess HNRNPA1 expression level. (B-C) Proliferation of EJ-1 and UM-UC-3 cells after HNRNPA1 knockout were analyzed using CCK8. (D) Cell growth of EJ-1 and UM-UC-3 cells after HNRNPA1 knockout were analyzed using clone formation assays. (E) Migration of EJ-1 and UM-UC-3 cells after HNRNPA1 knockout was assessed using wound healing, scale bar: 200 μm. (F) Invasion of EJ-1 and UM-UC-3 cells after HNRNPA1 knockout was assessed using Transwell invasion assay, scale bar: 20 μm. *$p < 0.05$, **$p < 0.01$, ***$p < 0.001$.

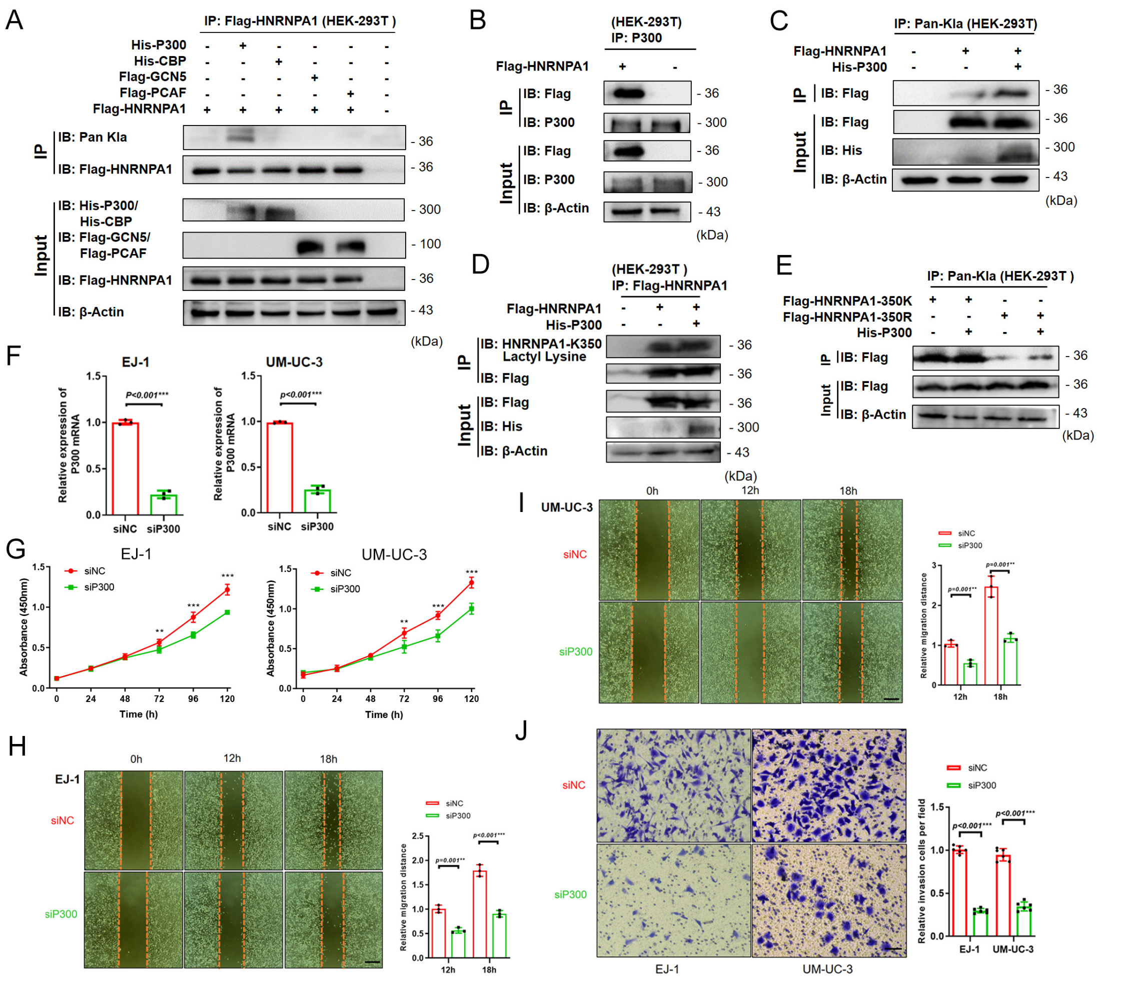

Supplementary Figure 7. P300 is the acyltransferase of HNRNPA1 K350 lactylation. (A) Lactylation of HNRNPA1 was determined by IP analysis. HEK-293T cells were transfected with plasmids expressing Flag-HNRNPA1 accompanied by overexpression of His-P300, His-CBP, Flag-GCN5 or Flag-PCAF. Equal amounts of lysates were prepared for IP with anti-Flag agarose beads, followed by detection of Pan-Kla. (B) HEK-293T cells were transfected with Flag-HNRNPA1 plasmid. Control IgG or anti-P300 antibody was used for IP followed by detection of Flag. (C) IP analysis of lactylation of HNRNPA1. HEK-293T cells were transfected with the indicated plasmids (His-P300 and Flag-HNRNPA1). Equal amounts of lysates were prepared for IP with anti-Pan-Kla antibody followed by detection of Flag. (D) IP analysis of HNRNPA1 K350 lactylation. HEK-293T cells were transfected with indicated plasmids (His-P300 and Flag-HNRNPA1) using anti-Flag agarose beads, followed by detection of HNRNPA1 K350 Lactyl Lysine. (E) IP analysis of lactylation of HNRNPA1. HEK-293T cells were transfected with the indicated plasmids or lentivirus (Flag-HNRNPA1 WT, Flag-HNRNPA1 K350R and His-P300). Equal amounts of lysates were prepared for IP with anti-Pan-Kla antibody, followed by detection of Flag. (F) P300 was detected

by qPCR. (G) Proliferation of bladder cancer cells was analyzed using CCK-8 assay. (H-I) Migration of bladder cancer cells was analyzed using scratch assay, scale bar: 200 μm. (J) Invasion of bladder cancer cells was analyzed using Transwell invasion assay, scale bar: 20 μm. All data are presented as mean ± SD. All data are presented as mean ± SD. *$p < 0.05$, **$p < 0.01$, ***$p < 0.001$.

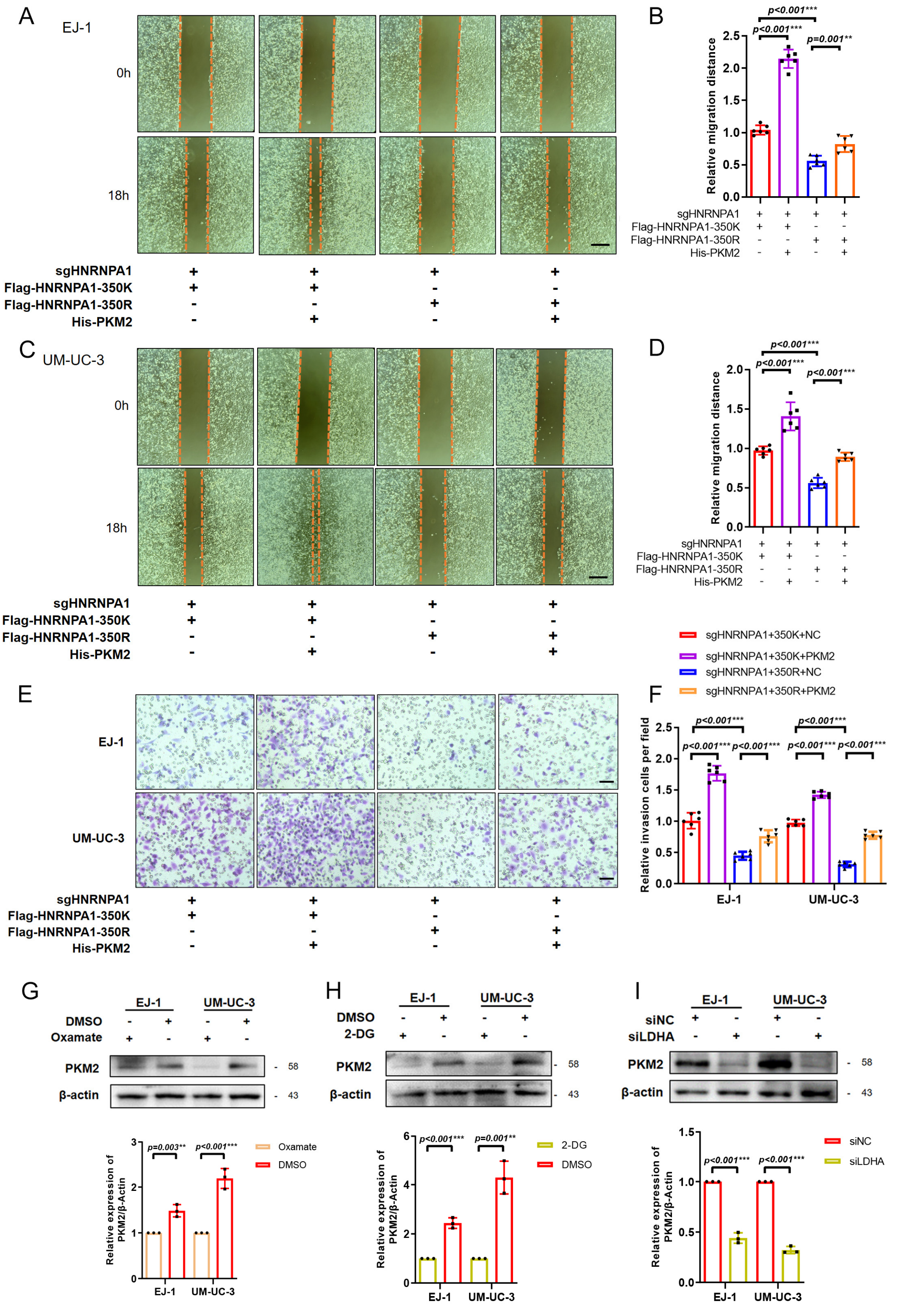

Supplementary Figure 8. HNRNPA1-K350 lactylation sustains aggressive phenotype via PKM2 splicing. (A-D) Wound-healing migration assay showing that PKM2 overexpression partially rescues the migration defect of HNRNPA1-K350R BLCA cells, scale bar: 200 μm. (E-F) Transwell invasion assay showing that PKM2 overexpression partially rescues the invasion defect

of HNRNPA1-K350R BLCA cells, scale bar: 20 μm. (G-I) Western blot was used to detect the PKM2 level in BLCA cells. All data are presented as mean ± SD. *$p < 0.05$, **$p < 0.01$, ***$p < 0.001$.

Supplementary Table 1. Clinical characteristics data

Patient No.	Age (y)	Gender (Female=0, Male=1)	Lactylation IHC Score (Tumor)	Lactylation IHC Score (Normal)	Histological Grade	T Stage	Overall Survival (y)	OS event (Alive=0, Death=1)
1	71	1	2.5	1	Low	T1	3.85	0
2	68	1	14	1	High	T1	2.13	0
3	74	0	2	1	Low	T1	0.39	0
4	78	0	14	4	High	T1	2.35	1
5	75	1	16	1	High	T4	0.52	1
6	69	1	14	1	High	T1	2.96	0
7	70	1	12	1	High	T1	0.12	1
8	73	0	16	0.5	High	T2	0.79	0
9	73	1	10.5	1	High	T1	1.69	1
10	79	0	12	0.5	High	T1	0.22	1
11	75	1	7.5	0	High	T1	0.49	0
12	86	0	6	1	Low	T2	4.87	0
13	66	1	16	1	High	T2	0.81	1
14	72	1	14	1	High	T3	3.31	0
15	70	1	10.5	0	High	T3	4.28	0
16	57	1	10.5	4	High	T2	4.48	0
17	57	1	1	1	Low	T1	3.52	0
18	77	1	11	4	High	T3	3.52	0
19	72	1	6	3	High	T3	0.73	1
20	66	1	14	0	High	T3	3.45	0
21	70	1	14	0.5	High	T3	3.5	0
22	78	1	2	2	High	T1	1.01	1
23	73	1	16	1	High	T3	0.85	0
24	54	1	10.5	1	High	T1	3.26	0
25	63	1	16	0.5	High	T2	0.93	1
26	70	1	16	1	High	T2	0.99	1
27	64	1	6	1	Low	T1	1.64	1
28	70	1	6	1.5	Low	T1	2.49	1
29	70	1	4	1	Low	T2	3.65	0
30	63	1	12	2	High	T2	0.51	1
31	73	1	1.5	1	Low	T2	2.36	0
32	76	1	10	1	High	T1	0.32	1
33	66	0	16	1	High	T3	1.81	1
34	58	1	12	1.5	High	T1	0.68	1
35	75	1	14	1	High	T1	3.5	0
36	65	1	12	1	High	T1	0.77	1
37	32	1	12	1	High	T2	1.31	1
38	75	1	1.5	4	Low	T1	4.18	0
39	71	1	12	1	High	T1	1.68	0
40	73	1	14	1	High	T3	0.67	1

41	57	1	2	1.5	Low	T1	1.46	1
42	56	1	9	1.5	High	T2	4.76	0
43	71	1	1	2.5	High	T1	3.92	0
44	69	1	2	3	High	T3	2.17	1
45	74	1	9	1	High	T1	4.8	0
46	75	1	16	1	High	T3	1.87	1
47	71	1	14	0	High	T2	3.39	0
48	70	1	16	1.5	High	T3	2.09	1
49	78	1	1.5	3	High	T3	4.04	0
50	86	0	7.5	1.5	Low	T2	3.06	1
51	68	1	6	1.5	High	T2	4.51	0
52	69	1	10.5	1	High	T3	3.42	0
53	74	1	14	1	High	T2	3.4	0
54	78	1	1.5	1	High	T1	0.14	0
55	78	1	9	1	High	T1	4.91	0
56	81	1	1.5	3	High	T3	0.38	0
57	72	1	10	1	High	T3	0.92	1
58	64	1	8	2	High	T1	4.26	1
59	63	1	3	4	Low	T1	3.98	0
60	64	1	3	3	High	T1	4.69	1
61	58	1	3	1	High	T1	3.15	0
62	65	0	9	1	High	T1	0.3	1
63	74	0	12	1.5	High	T1	1.7	1
64	50	1	14	2	High	T3	3.41	0
65	59	1	7.5	1	High	T1	2.22	1
66	89	1	12	1	High	T4	4.71	0
67	73	1	5	1.5	High	T1	4.07	0
68	68	1	7	4	High	T2	4.84	0
69	68	1	6	2	Low	T1	4.64	0
70	63	1	4	1	High	T3	2.6	0
71	70	1	4	1	Low	T1	2.67	1
72	74	1	5	1	Low	T1	3.84	0
73	75	1	16	1	High	T2	2.39	0
74	76	1	10.5	1	High	T2	3.49	0
75	52	1	8	4	High	T2	3.76	0
76	59	1	1	1.5	Low	T1	1.06	0
77	60	1	6	1	High	T2	3.78	0
78	62	1	1	1	High	T2	3.24	0
79	76	1	4	2	High	T2	1.2	0
80	58	1	4	1.5	Low	T1	0.35	1
81	74	1	8	1	High	T1	3.96	0
82	73	1	5.5	1.5	High	T3	4.02	0
83	68	1	4	8	High	T2	0.69	1
84	72	1	8	8	High	T2	3.99	0

85	61	1	10.5	1	High	T3	3.5	0
86	61	1	10	1	High	T3	1.39	0

Supplementary Table 2. Plasmids and siRNA used in this study

PLASMID	SPECIES	TAG	VECTOR	IDENTIFIER	SOURCE
P300	Human	6×His	pcDNA3.1	G0316073	General Bio (China)
CBP	Human	6×His	pcDNA3.1	G0315422	General Bio (China)
PCAF	Human	3×Flag	pcDNA3.1	H21376	Obio (China)
GCN5	Human	3×Flag	pcDNA3.1	H24038	Obio (China)
HNRNPA1	Human	3×Flag	pcDNA3.1	R8314	YHDURO (China)

siRNA	SPECIES	TARGET SEQUENCE	SOURCE
siLDHA	Human	CUCUAAAGGAUCAGCUGAUTT	General Bio (China)
siP300	Human	CAGAGCAGUCCUGGAUUAGTT	General Bio (China)

Supplementary Table 3. Reagents used in this study

REAGENT	IDENTIFIER	SOURCE
Lactate sodium (NaLa)	Cat#867-56-1	Sigma Aldrich (USA)
Oxamate	Cat#HY-W013032A	MedChemExpress (USA)
2-DG	Cat#HY-13966	MedChemExpress (USA)
CTB (P300 activator)	Cat#HY-134964	MedChemExpress (USA)
C646(P300 inhibitor)	Cat#HY-13823	MedChemExpress (USA)
Polydatin	Cat#27208-80-6	TargetMol (USA)
Insulin	Cat#P3376	Beyotime (China)
Cell Counting Kit-8	Cat#C6005	New Cell & Molecular Biotech (China)
Crystal violet	Cat#C0121	Beyotime (China)
EdU Cell Proliferation Kit	Cat#C10310	Ribobio (China)
Triton X-100	Cat#GC204003	Servicebio (China)
Matrigel	Cat#354230	Corning (USA)
Annexin V-FITC/PI Apoptois Kit	Cat#40302ES	Yeason (China)
IP lysis buffer	Cat#P70100	New Cell & Molecular Biotech (China)
Protein A/G agarose beads	Cat#sc-2003	Santa Cruz (USA)
DAB Kit	Cat#ZLI-9018	ZSGB-Bio (China)
Fetal Bovine Serum	Cat#FSS500	Excell (China)
RPMI-1640 medium	Cat#11875101	Gibco (USA)
F12K medium	Cat#21127022	Gibco (USA)
Hematoxylin	Cat#G1080	Solarbio (China)
Glucose Uptake Assay Kit	Cat#ab136955	Abcam (UK)
L- Lactate Assay kit	Cat#ab65331	Abcam (UK)
ATP Assay Kit	Cat#S0026	Beyotime (China)
Extracellular Acidification Rate Kit	Cat#103010-100	Seahorse Biosciences (USA)
$NADP^+$/NADPH assay	Cat#S0179	Beyotime (China)
GSH/GSSG assay	Cat#S0053	Beyotime (China)
D-Glucose Content Assay Kit	Cat#AKSU001M	Boxbio Science&Technology (China)
HitransG A Transfection Reagent	Cat#REVG004	GeneChem (China)
X-tremeGENE HP DNA Transfection	Cat#06366244001	Roche (Switzerland)

Supplementary Table 4. Antibody used in this study

ANTIBODY	IDENTIFIER	SOURCE
Anti-HNRNPA1	Cat#sc-32301	Santa Cruz (USA)
Anti-LDHA	Cat#2012s	Cell Signaling Technology (USA)
Anti-β-actin	Cat#SDT-R015	Starter Biotechnology (China)
Anti-HNRNPA1 K350 Lactyl Lysine	Cat#COPTM-698	PTMBIO(China)
Anti-Pan Kla	Cat#PTM-1401	PTMBIO(China)
Anti- P300	Cat#sc-32244	Santa Cruz (USA)
Anti-Flag	Cat#20543-1-AP	Proteintech (China)
Anti-His	Cat#GB151255-100	Servicebio (China)
Anti-PKM1	Cat#15821-1-AP	Proteintech (China)
Anti-PKM2	Cat#15822-1-AP	Proteintech (China)
HRP-conjugated Goat Anti-Rabbit IgG	Cat#ab97051	Abcam (UK)
HRP-conjugated Goat Anti-Mouse IgG	Cat#RS020209	Immunoway (USA)
Normal rabbit IgG	Cat#2729	Cell Signaling Technology (USA)
Normal mouse IgG	Cat#B900620	Proteintech (China)

Supplementary Table 5. Primer used in this study

Primer		Sequence (5'-3')
LDHA	Forward	TTGACCTACGTGGCTTGGAAG
	Reverse	GGTAACGGAATCGGGCTGAAT
P300	Forward	GATGACCCTTCCCAGCCTCAAA
	Reverse	GCCAGATGATCTCATGGTGAAGG
PKM1	Forward	CCCACTCGGGCTGAAGGCAGTG
	Reverse	GCTGCCTCAGCCTCACGAGC
PKM2	Forward	CCCACTCGGGCTGAAGGCAGTG
	Reverse	GGCAGCCTCTGCCTCACGGG
β-actin	Forward	CAAACCGCTGCTCAATCTTC
	Reverse	AGTTTGGTCAATACCGGCAG

Supplementary Table 6. Lactylation-related genes used for single-cell analysis

AARS1	LDHA
AARS2	LDHB
CREBBP	LDHC
EP300	SIRT1
ESCO2	SIRT2
HDAC1	SIRT3
HDAC2	SIRT6
HDAC3	SLC16A1
HDAC8	SLC16A4
KAT2A	SLC16A7
KAT5	SLC5A12
KAT7	SLC5A8
KAT8	SMARCA4
